# Bioprinted Ventilated 3D Alveoliform Epithelial Sacculoids

**DOI:** 10.21203/rs.3.rs-8177180/v1

**Published:** 2025-12-30

**Authors:** Ibrahim Ozbolat, Myoung Hwan Kim, Joseph Moses, Zissis Chroneos, Todd Umstead, Mian Horvath, Candace Chan, Julia Oh

**Affiliations:** The Pennsylvania State University; The Pennsylvania State University; The Pennsylvania State University; Penn State University College of Medicine; Penn State University College of Medicine; University of Connecticut Health; Penn State University College of Medicine; Duke University School of Medicine

**Keywords:** Bioprinting, Organoids, Lungs, Alveolar Sac, Alveolar Epithelial Cells, Development, Disease Model, Ventilation, Mechanotransduction

## Abstract

Understanding the human distal lung requires ex-vivo models that capture both the structural hierarchy and mechanical environment of alveolar sacs, yet current ex-vivo systems fall short. Manually cultured organoids and two-dimensional cell cultures lack structural hierarchy, apical access, and physiologic actuation via ventilation, limiting their use in modeling infection and mechano-transduction. Here, we established three-dimensional (3D) alveolar epithelial sacculoids (AES) by bioprinting pluripotent stem cell-derived alveolar epithelial type II cells (ATIIs) into defined 3D geometries in high density. AES reproducibly self-organized into multi-unit, lumenized sacs with polarized epithelia, surfactant secretion, and functional heterogeneity including proliferative ATIIs, surfactant-producing ATIIs, and transitional pre-alveolar type I transitional cell state (PATS)-like cells. A custom air-driven platform enabled fluid-mediated 3D ventilation, producing volumetric oscillations across closed sacs. This actuation engaged canonical (Ser127) and non-canonical (Tyr357, integrin–FAK–dependent) Hippo signaling, driving ATII-to-ATI remodeling and junctional stabilization. Upon apical infection with influenza virus, AES recapitulated canonical antiviral responses and epithelial plasticity resembling injury-induced alveolar repair, including depletion of functional ATIIs and emergence of proliferative ATIIs and transitional states. AES represent a physiologically ventilated model of the human alveolar niche, enabling mechanistic studies of epithelial plasticity, viral pathogenesis, and biomechanical signaling under physiologically-relevant conditions. Overall, our model provides a foundation for future integration of stromal, vascular, and immune components toward full alveolar mimicry to facilitate ex-vivo translational respiratory research.

## Introduction

Alveolar sacs consist of several intricately organized three-dimensional (3D) alveoli clusters that are interconnected to facilitate gas exchange through cyclical expansion and contraction during respiration. In addition to its gas exchange role, alveolar sacs also provide an important role of maintaining barrier integrity and mediating innate immune responses against pathogenic entry of foreign particles through their epithelium lining^1–3^. Two specialized epithelial cell types dominate this niche: flat alveolar type I cells (ATIs), which constitute the majority of the alveolar lining and form the thin air–blood barrier, and cuboidal alveolar type II cells (ATIIs), which serve as facultative progenitors of the alveoli^3,4^. ATIIs secrete surfactant proteins and lipids to reduce surface tension and prevent alveolar collapse, while also acting as immune sentinels that express pattern-recognition receptors, orchestrating host defense and coordinating repair following injury in concert with resident macrophages.

The global impact of COVID-19 pandemic has highlighted the importance of understanding how respiratory viruses interact with lung tissue, particularly the alveolar epithelium. In the event of an injury (such as viral infection), ATIIs can self-renew and differentiate into ATIs to restore epithelial continuity after damage. While much of our understanding of this ATII→ATI plasticity has been derived from lineage-tracing studies in mouse models^5–8^, these systems have inherent limitations, particularly in modeling human-specific features, such as viral tropism and pathogen entry. Though mice offer unique advantages of genetic-labelling specific alveolar epithelial progenitors to track their differentiation and contribution to repairing the alveolar lining after injury, they do not recapitulate the cell-type specific susceptibility and infection pathways characteristic of the human alveoli. This understanding is essential for advancing therapeutic strategies to prevent future pandemics against infections and projects the need for robust, biomimetic 3D ex-vivo human models to capture the unique biology of the alveolar epithelium and its role in injury, repair, and host-pathogen interactions.

Most efforts to model the alveolar epithelium primarily involve alveolar epithelial cells cultured on two-dimensional (2D) porous (Transwell inserts) or micropatterned substrates^8–11^, or within microfluidic platforms^12–14^. While these systems have advanced our understanding of epithelial biology and infection dynamics, these reductionist systems do not recapitulate the dynamic interplay between ATIs and ATIIs that drives alveolar homeostasis and repair. They insufficiently replicate the 3D architecture of the alveoli, where interconnected sac-like compartments and dynamic epithelial organization are essential to accurately simulate post-natal lung repair, disease progression, and responses to physiological ventilation. Among diverse cell sources tested for alveolar modeling, including immortalized cell lines and primary ATIIs, lung organoids derived from the directed differentiation of induced pluripotent stem cells (iPSCs) into ATIIs has emerged as a robust strategy^15^. Unlike primary ATIIs, which rapidly lose proliferative capacity *ex vivo*, iPSC-derived ATIIs can self-organize into alveolospheres within 3D basement membrane matrices, self-renew and maintain surfactant processing and innate immune programs, and offer genetically tractable, patient-specific models^15^. Although these single alveolosphere-based organoid systems advance biological relevance over traditional cultures, alveolospheres pose limitations with inconsistent size and morphology across passages, making quantitative comparisons challenging. To mimic breathing, existing models often rely on cyclic substrate stretch, whereas physiologically relevant ventilation systems remain rare and often impose non-physiological shear stress, epithelial over-distension, and impaired surfactant dynamics^8,16–18^. Moreover, infection studies often expose viral particles to basal rather than apical surfaces and alveolospheres lack the complex structural integrity and mechanical stimuli inherently present in the native alveolar microenvironment.

Here, we introduce engineered alveoliform epithelial sacculoids (AES) as ATII-derived, mesenchyme-free, lumenized epithelial constructs that self-organize into interconnected, budding alveoliform units with (i) defined apico-basal polarity, (ii) the pores of Kohn-like apertures allowing communication between adjacent alveoli, (iii) apical accessibility for infection studies, and (iv) compatibility with controlled ventilation. By precisely depositing iPSC-derived ATIIs in high density into Matrigel (henceforth referred to as ‘Mat’), we achieved reproducible sacs with consistent size. These ATIIs self-organized into alveoli-like structures, wherein budding occurs via epithelial remodeling without mesenchymal induced septation or the inclusion of endothelial, stromal, or immune cells, while exhibiting surfactant secretion, epithelial polarity, and morphogenetic remodeling. Applying cyclic mechanical stretch through positive pressure-driven 3D ventilation revealed mechanotransduction programs and ATII plasticity. We further evaluated physiological functionality by apically infecting AES with influenza A (PR8 H1N1) and profiling molecular and transcriptomic host-pathogen responses. This ex-vivo platform provides a reproducible, human-relevant epithelial alone model to interrogate distal epithelial lung biology, post-natal regeneration, mechanically driven epithelial plasticity, and infection dynamics.

## Results

### 1. ATIIs bioprinted in high density with positional precision drive reproducible sac-like formation.

**Figure 1:**
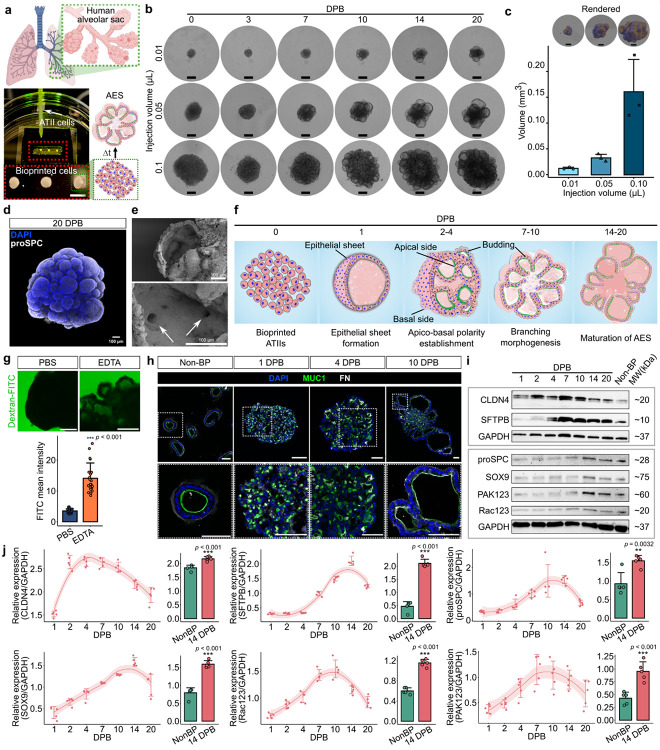
Stepwise morphogenesis of AES. a, Schematic illustration of the human distal lung alveolar sac (created in BioRender) and bioprinted iPSCs-derived ATIIs for AES development. b, Representative images of precisely patterned ATIIs with varying cell cluster injection volumes showing size-controllable AES formation and their morphological progression over time. Scale bar: 200 μm. c, 3D reconstruction of AES generated from different injection volumes with corresponding volumetric measurements (*n* = 3 independently prepared AES). Scale bar: 200 μm. d, Whole-mount immunostaining of demonstrating proSPC expression throughout the AES structure. e, Representative SEM images showing cross-section of an AES and the presence of pore-like structures (indicated by white arrows) resembling pores of Kohn within AES. f, Illustration summarizing sequential steps of AES morphogenesis and development, including epithelial sheet formation, apico-basal polarization, and structural remodeling at respective days post bioprinting (DPB). g, Barrier integrity measurement of AES comparing PBS-treated controls and EDTA-destabilized AES using 4 kDa FITC-Dextran. Right, quantification of FITC intensity located inside of AES (*n* = 22, independently prepared AES). Scale bar: 100 μm. h, Immunostaining of MUC1 and FN, demonstrating progressive establishment of apico-basal polarity; nuclei stained with DAPI. Scale bar: 100 μm. i, Representative immunoblots of CLDN4, SFTPB, proSPC, SOX9, PAK123, and Rac123 expression during AES development. Blots were representative of 5 independently performed experiments with similar results. Non-cropped full blots were included in Fig. S15. j, Quantification of protein expression changes during AES development by densitometry normalized to GAPDH (*n* = 5). Data were presented as mean ± SD, where *p < 0.05, **p < 0.01, and ***p < 0.001.

After the differentiation of iPSCs into ATIIs using an established protocol^15^ (**Fig. S1a** and **Supplementary Note**), ATIIs were maintained in Mat in 3D, where manual embedding of ATIIs in Mat occasionally yielded bundled alveolospheres in regions of high local cell density, suggesting that packing ATIIs in high density promotes alveolar morphogenesis (**Fig. S1b, green arrow**). To better facilitate such a condition, we employed a bioprinting approach to precisely deposit ATIIs through a pulled glass nozzle into pre-gel Mat loaded in custom-made devices with controlled volume and spatial positioning (Figs. 1a and **S2**). We found that the residual culture medium of ATIIs in the glass nozzle disrupted uniform deposition by introducing density gradients within the nozzle. To evaluate the effect of residual medium, ATII pellets were prepared in three conditions: minimal residual medium (< 5 μL), and suspensions containing 100 or 500 μL of the medium (**Figs. S2a** and **S2b**). Each group was bioprinted in 0.1 μL injection volumes using a syringe pump and monitored for AES formation. Pellets prepared with minimal residual medium (<5 μL, **Fig. S2aiii**) produced homogeneous extrusion, uniform ATII distribution, and a 100% efficiency of AES formation within 10 days post bioprinting (DPB) (**Fig. S2c**). In contrast, pellets containing 100 or 500 μL of medium showed inconsistent morphogenesis, reflecting impaired cell-cell interactions and heterogeneous dispersal (**Fig. S2c**). While the residual medium was difficult to remove completely, our experiments revealed that below 5 μL allowed successful loading of highly packed cell pellets. Importantly, neither the total cell number in the pellet nor the nozzle diameter affected bioprinting efficiency, as gravitational settling ensured consistent pellet packing and syringe-pump injection volumes remained tightly controlled. To assess the precision of cellular deposition via bioprinting, ATIIs were deposited at defined injection volumes ranging from 0.001 to 0.5 μL per spot. Quantification of deoxyribonucleic acid (DNA) and total protein content showed a linear rise with increasing injection volume, demonstrating the feasibility to finely control cell density and deposition size (**Figs. S3a** and **S3b**). Cell viability remained >99% before and after extrusion, indicating that the process preserved cellular integrity and did not cause any major shear stress-induced cell damage during extrusion (**Fig. S3c**).

We next bioprinted ATIIs across selected injection volumes (0.01, 0.05, 0.1 μL) (**Supplementary Movie 1**) and assessed morphogenesis of AES (Fig. 1b). Immediately after bioprinting (0 day post bioprinting (DPB)), cells formed compact clusters proportional to the injection volume. By 7 DPB, ATIIs self-organized into epithelial sacculoids with well-defined boundaries, which progressively stabilized and expanded in size. Quantitative 3D reconstructions showed AES volumes scaled directly with injection volume: 0.012 ± 0.003 mm^3^ (0.01 μL), 0.032 ± 0.007 (0.05 μL) and 0.16 ± 0.063 mm^3^ (0.1 μL), respectively (Fig. 1c). Engineered AES (0.16 ± 0.063 mm^3^ in volume), corresponding to ~40 alveoli (considering the volume of a single human alveolus to be ~0.004 mm^3 19^), represent the size range that most closely approximates native alveolar sacs and were therefore used for subsequent studies. More importantly, these AES displayed interconnected alveolar compartments akin to native human alveolar sacs. In the developed AES, NKX2–1^+^ progenitors (illustrated in **Supplementary Note** and **Fig. S1e**) predominated throughout the structure, demonstrating the spatial distribution of lung progenitor populations and internal architectural organization (**Supplementary Movie 2**). Whole-mount confocal imaging further confirmed that AES maintained polarized cuboidal epithelium and robust proSPC expression at 20 DPB and exhibited intricately budded morphogenesis resembling the native structure (Fig. 1d). We identified pore-like structures in the inter-alveolar septa of cross-sectioned AES, resembling the pores of Kohn in native alveolar sacs, which enable interconnection between adjacent alveoli *in vivo* (Fig. 1e and **Supplementary Movie 2**). These pores measured approximately 8–15 μm in diameter, closely matching the average pore size range observed in adult human alveolar sacs (7–19 μm)^20,21^.

### 2. Intrinsic epithelial programs govern AES formation.

High-density ATIIs self-organize into epithelial sheets that acquire apicobasal polarity and lumenize into sac-like units, recapitulating a conserved epithelial morphogenesis program in a mesenchyme-free context (Fig. 1f). While native postnatal alveologenesis relies on stromal mechanics (myofibroblast elastin rings and endothelial basement-membrane scaffolds), our system depicts the epithelial component of sac formation and reveals its sufficiency for polarity-driven alveolar morphogenesis. To assess epithelial barrier integrity, AES (20 DPB) were immersed in an 4-kDa fluorescein isothiocyanate (FITC)-labeled Dextran solution to evaluate paracellular leakage (Fig. 1g). Under normal conditions, the Dextran signal was not detected in the lumen, indicating a tight epithelial barrier. In contrast, the treatment with ethylenediaminetetraacetic acid (EDTA), a chelator to disrupt adherens junctions, resulted in compromised cell-to-cell integrity and clear infiltration of Dextran into the lumen). This confirms the presence of intact and functional epithelial barrier in bioprinted AES demonstrating that they recapitulated not only the structural hallmarks of native alveoli but also the functional barrier integrity essential for physiological relevance.

#### 2.1 Epithelial polarity precedes functional maturation during alveolar sac morphogenesis.

We hypothesized that high-density, spatially constrained ATIIs would self-organize into AES via intrinsic polarization and epithelial sheet remodeling. Mat, which is rich in basement membrane proteins, such as laminin, collagen IV, and arginyl-glycyl-aspartic acid (RGD) peptides^22^, supports epithelial organization by engaging integrin receptors, including α3β1, α6β4, to activate integrin-linked kinase signaling pathways that promote cell polarization and epithelial integrity^23^. Within Mat, β-integrin receptors on the ATII surface interact with RGD peptides to initiate coordinated global apicobasal polarization and epithelial sheet remodeling^23^. To track the polarization dynamics, we stained AES for mucin-1 (MUC1, an apical marker^24^) and fibronectin (FN, a basal surface marker^25^) (Fig. 1h). At 1 DPB, bioprinted ATIIs compacted and began forming cohesive epithelial sheets. By 7 DPB, a well-organized epithelial layer exhibiting distinct apicobasal polarity was established, with clear spatial segregation of MUC1 and FN (Figs. 1h and **S4**). Temporal profiling revealed that Claudin-4 (CLDN4, a tight-junction protein) was highly expressed during early sheet formation, consistent with tight junction assembly, but declined as AES transitioned into polarized states (Figs. 1i and 1j)^26^. Polarity-driven epithelial remodeling involved distinct morphological changes during the developmental program, including elongation, reconnection, and reconstitution between apical and basal surfaces. Polarity-driven epithelial remodeling initiated alveolar morphogenesis, giving rise to nascent, polarized alveolar-like compartments by 7 DPB through planar cell polarity (PCP) signaling. PCP, which governs lateral alignment across epithelia, coordinated barrier function, epithelial sheet orientation, and morphogenetic behavior^27^. The mutual inclusion or exclusion of the regulatory factors in PCP signaling cascades, particularly the atypical cadherin (FAT / Dachsous cadherin-related protein (DCHS) cascade), create an asymmetry in the developed epithelium, thereby sensing extrinsic signals from neighboring cells and the local microenvironment (such as RGD/β-integrin mediated signaling) with polarity-regulated signaling^28,29^. In parallel, the progenitor regulator SRY-box transcription factor 9 (SOX9) and cytoskeletal effectors Rac and p21-activated kinase (PAK) showed transient upregulation, reflecting their roles in epithelial compaction and morphogenetic remodeling (Figs. 1i and 1j)^30^. Recent studies reveal that in fetal ATII organoid models, epithelial progenitors undergo maturation only after establishment of polarity^31^. Our results, which show early upregulation of Rac and PAK coincide with epithelial sheet formation and prior to surfactant expression, align with these observations and extend them by delineating a timeline in a mesenchyme-free, bioprinted system. When directly compared at 14 DPB to non-bioprinted alveolospheres (NonBP, as described in^15^), AESs exhibited significantly higher expression of SOX9, Rac, PAK, SFTPB, and proSPC (Fig 1j), highlighting accelerated progression from structural remodeling toward functional competence (maturity) in terms of surfactant production. Importantly, after the apicobasal polarity was established, SFTPB expression progressively increased with sac maturation, marking the transition from structural remodeling to functional competence.

#### 2.2 Dynamic pMerlin localization coincides with proliferative remodeling and AES maturation.

**Figure 2:**
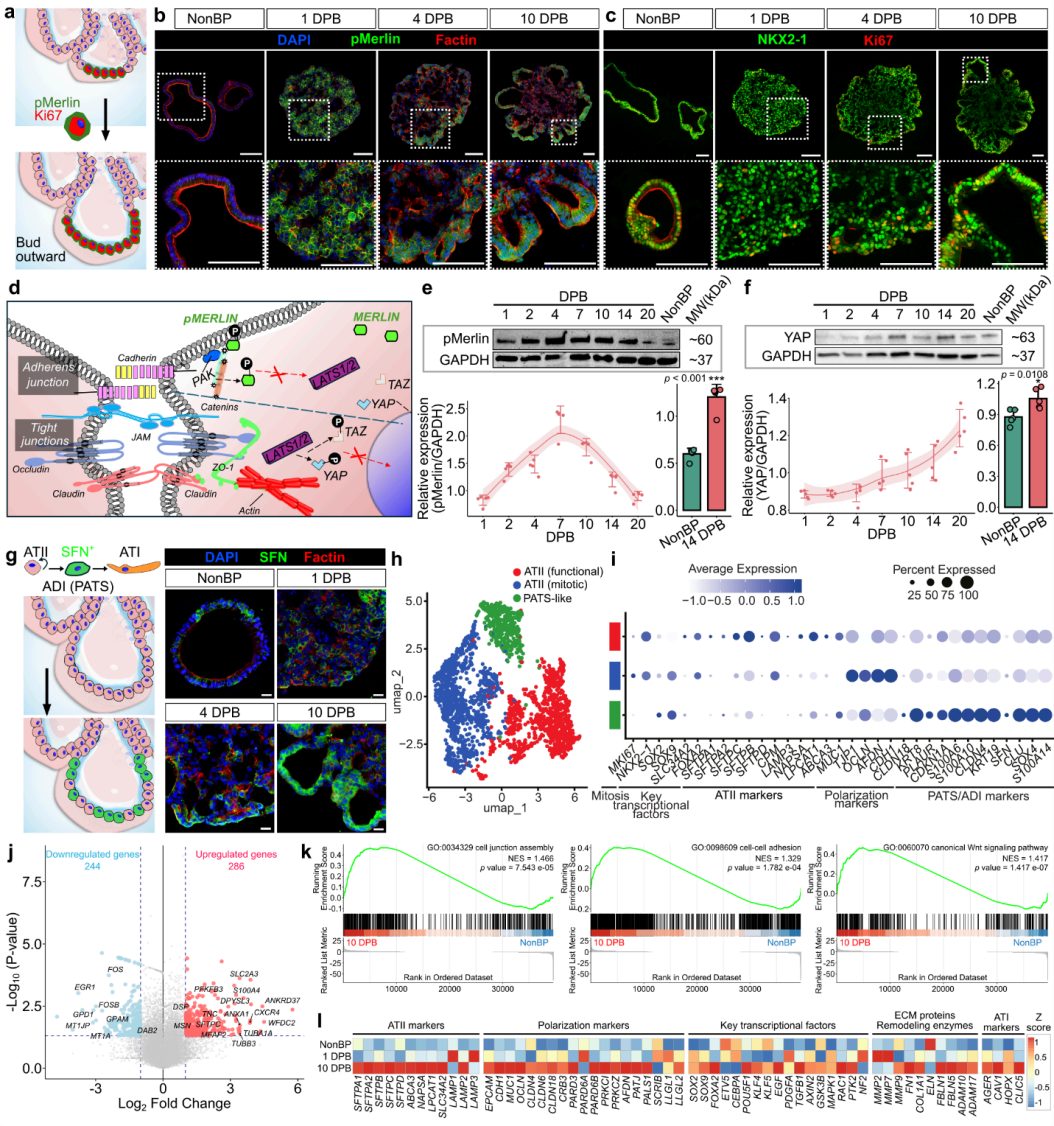
Peripheral Budding and Lineage Progression in AESs. a, Schematic representation showing peripheral budding driven by pMerlin and proliferative Ki67^+^ cells during AES development. b, Immunostaining of pMerlin demonstrating its progressive enrichment and peripheral localization in AES; nuclei were stained with DAPI and the cytoskeleton with F-actin. Scale bar: 100 μm. c, Immunostaining of NKX2–1 and Ki67 showing proliferative activity at the periphery, indicating outward budding regions with enriched lung progenitors during AES morphogenesis. Scale bar: 100 μm. d, Schematic illustration of Merlin phosphorylation at cell junctions, leading to modulation of the Hippo signaling pathway. e-f, Representative immunoblots showing expression dynamics of (e) pMerlin and (f) YAP during AES development, with their densitometric quantification normalized to GAPDH (*n* = 5). Blots were representative of 5 independent experiments with similar results. Non-cropped full blots were included in Fig. S16. g, Immunostaining of SFN demonstrating its progressive enrichment and peripheral localization in AES as transitionary phenotype. h, UMAP visualization of single-cell transcriptomes from NonBP and bioprinted groups (14DPB). i, Dot plot depicting expression of key ATII gene markers, including: mitosis, key transcriptional factors, ATII markers, polarization markers, and PATS/ADI markers. j, Volcano plot of DEGs between NonBP and AES populations. k, GSEA revealing significantly enriched biological pathways associated with AES development. i, Heatmap of representative DEGs highlighting transcriptional changes over time. Data were presented as mean ± SD, where *p < 0.05, **p < 0.01, and ***p < 0.001.

To probe the molecular mechanisms linking polarity to morphogenesis, we focused on Merlin / neurofibromin 2 (NF2), a regulator of cytoskeletal dynamics and intercellular communication^32,33^. Junctional complexes are known to integrate extracellular cues with cytoskeletal responses to maintain polarity and drive epithelial remodeling^34^. We hypothesized that phosphorylated Merlin (pMerlin; phosphorylation mediated through PAK^35^), a member of ezrin/radixin/moesin signaling (ERM) axis, acts as a mechanochemical transducer regulating alveolar sheet remodeling. Phosphorylation of Merlin at ser-518 (pMerlin) converts Merlin to an inactive form, thereby weakening Hippo pathway activity and permitting Yes-associated protein (YAP) nuclear entry^36^. In our ex-vivo model, pMerlin functions as a mechanochemical transducer: its dynamic localization at epithelial boundaries supports local proliferation and outward budding, while downstream Hippo/YAP signaling coordinates remodeling and lineage progression^37,38^. At 1 DPB, pMerlin was broadly expressed throughout both central and peripheral regions of the forming epithelial sheet, with clear localization at cell membranes, indicative of active cell-to-cell interactions and early morphogenic remodeling (Figs. 2a–2b and **S5a**). pMerlin became enriched at the periphery and budding fronts, where Ki67^+^ (a marker of cellular proliferation) cells clustered in NKX2–1^+^ ATIIs (Figs. 2a–e and **S5b**), consistent with the role of pMerlin in licensing localized growth and epithelial sheet remodeling. This is in line with the established antagonism reported between Merlin and YAP in developing epithelia^39^. YAP levels and nuclear localization rose over time (Fig. 2f), matching the expectation that Hippo attenuation as evidenced by an increased YAP activity supports alveolar repair and lineage progression^40^. Importantly, when examined head-to-head at 14 DPB, AES exhibited stronger YAP activation relative to NonBP (alveolospheres), suggesting that the engineered 3D microenvironment enhances mechanosensitive Hippo-YAP signaling during sac remodeling.

As AES morphogenesis progressed, bioprinted ATIIs exhibited increasing complexity and size during sac development. By 10 DPB, cells within AES compartments began to express stratifin (SFN, a well-established marker of PATS cells), which have the potential to differentiate into ATIs (Fig. 2g)^41,42^. SFN expression localized predominantly to apico-basally polarized regions of the alveolar epithelium, particularly at structurally mature AES exhibiting luminal expansion and epithelial sheet stabilization. These findings suggest that high-density, bioprinted ATIIs not only underwent sheet remodeling and morphogenesis, but also transitioned into an intermediate PATS phenotype, reflecting in-vivo alveolar maturation processes and positioning our model to support further ATII-to-ATI lineage transitions via YAP-linked transitional programs (SFN^+^ alveolar differentiation intermediate (ADI) / PATS). Pertinent to note here, the decline in SOX9 at later stages (Figs. 1i and 1j) reflects a shift toward polarity-driven differentiation programs, reinforced by dynamic Merlin-YAP signaling (Fig. 2f) and emergence of SFN^+^ transitional states, rather than loss of epithelial viability. To rule out the possibility that declining SOX9 reflected sac degeneration at later stages, we assessed cell survival. TUNEL staining revealed only transient apoptosis at early time points (2–4 DPB), which resolved as AES matured over time post bioprinting (**Figs. S6a** and **S6b**). Consistently, levels of BAX (BCL2-associated X protein), a pro-apoptotic member of the BCL2 family, peaked during early remodeling but declined thereafter, where the pro-survival factor BCL2 (B-cell lymphoma 2) increased by 14–20 DPB (**Fig. S6c**). These results confirm that bioprinted AES remained viable and structurally intact during late maturation, supporting that SOX9 downregulation (Fig. 1j) reflects differentiation rather than cell death.

#### 2.3 Single-cell and bulk transcriptomic profiling identify enhanced structural and functional pathways in AES

To portray the cellular heterogeneity within AES, we performed single-cell ribonucleic acid sequencing (scRNA-seq) of bioprinted AES (14 DPB), where NonBP was used as a control. Following integration, three clusters were identified as canonical ATII markers^15,43–45^ (Figs. 2h and 2i): mitotic ATII (*MKI67*), functional ATII expressing surfactant proteins (*SFTPA1*, *SFTPA2*, *SFTPB, SFTPC*, and *SFTPD*) and *MUC1*, and PATS-like cells expressing genes previously described to characterize PATS^42^ and KRT8^+^ alveolar differentiation intermediate (ADI)^46^ populations (*KRT8*, *PLAUR*, *CDKN1A*, *S100A6*, *S100A10*, *CLDN4*, *KRT19*, *SFN*, *CLU*, *SOX4*, *S100A14*).

Pathway enrichment of deferential gene expression (DEG) revealed distinct programs across clusters (**Figs. S7a**-**c**). Functional ATIIs overexpressed structural junctional modules (tight junctions, adherens junctions, focal adhesion) and morphogenetic signaling pathways (Hippo, MAPK, PI3K-Akt, Hedgehog, FoxO) (**Fig. S7a**). This aligns with our observation that epithelial polarity precedes surfactant maturation. Mitotic ATIIs were enriched in biosynthetic and metabolic pathways (nucleotide metabolism, glycolysis, N-glycan biosynthesis), consistent with proliferative expansion (**Fig. S7b**). PATS-like cells showed stress-responsive and transitional programs (HIF-1, FoxO, ferroptosis), echoing prior reports that ATIIs can adopt a transient “stressed progenitor” fate enroute to differentiation (**Fig. S7c**)^42^. Altogether, bioprinting biases the epithelial program toward structural/polarity modules and unmasks a transitional ADI/PATS-like state on the path to functional ATII maturation, aligning with and extending published single-cell maps to a mesenchyme-free^44^, structurally controlled alveolar sac model.

To assess the durability of AESs, we profiled transcriptional dynamics across passages (denoted as P#) (**Fig. S8**). Up to P17, both AES and NonBP alveolospheres maintained key ATII features, but a decline was noted after P17 in NonBP as well as 10 DPB AES. We next investigated how prolonged passaging affected the transcriptional stability of AES. Heatmap analysis of curated senescence and EMT (epithelial to mesenchymal transition) associated markers showed a clear passage-dependent shift: while both systems (NonBP and AES) maintained robust ATII identity up to ~P17. However, later passages (≥P19) displayed induction of senescence-associated genes (SASP) (*TNF, CXCL2, IGFBP3/4, GDF15, MMP7/10/12, CDKN2A*) (**Fig. S8**). These signatures suggest progressive senescence and EMT onset with extended culture^47^. Importantly, AES delayed these changes compared to the NonBP control, consistent with enhanced epithelial stability. AES bioprinted with ATIIs (<P19) exhibited transcriptional features aligned with our morphogenetic framework. Volcano plots (Fig. 2j) also revealed upregulation of genes supporting epithelial sheet formation and polarity including *DSP* (desmosomal plaque protein)^48^, *TUBA1A*/*TUBB3* (microtubule isoforms supporting planar cell polarity)^49^, *DPYSL3* (actin bundling), and *MSN* (ERM family, Merlin-interacting). Additional hits such as *TNC* (tenascin), *MFAP2* (microfibril-associated), and *CXCR4* (C-X-C motif chemokine receptor 4) pointed to integrin/ECM (extracellular matrix) signaling^50^ and Rac/MMP (matrix metalloproteinase)-driven epithelial remodeling. Functional ATII programs were reinforced by *S100A4*, *ANXA1* (annexin A1), surfactant protein (*SFTPC*), and metabolic rewiring toward glycolysis (*PFKFB3*, *SLC2A3*). Conversely, stress-response genes (*EGR1/FOS/FOSB*, *MT1JP*, *DAB2*)^51,52^ and fibrosis-associated adaptors (*PI3K*–*TGF-β* amplifiers) were downregulated, suggesting reduced oxidative stress burden and absence of profibrotic signaling in AES. Gene set enrichment analysis (GSEA) further validated these patterns: AES (from <P19) were enriched in actin cytoskeleton organization, cell adhesion, junction assembly, canonical Wnt signaling, and ECM organization pathways (Figs. 2k and **S7d**). The transcriptional profile (**Fig. S8**) confirmed that up to P17 undergo polarity-driven remodeling, junctional strengthening, and morphogenesis toward functional maturation (in terms of polarity and surfactant production). In contrast, AES from later passages (≥P19) revealed overt transcriptional deterioration. Heatmaps demonstrated widespread upregulation of EMT markers (*VIM, COL1A1/3A1, TGFB1/2, SMAD3*) (**Fig. S8**). Importantly, polarity-associated markers (*CLDN4, MUC1*) that were preserved up to P17 became progressively downregulated, indicating loss of epithelial barrier function. Volcano plots showed strong induction of *OLFML3* (ECM glycoprotein, EMT onset), *IGFBP5* (collagen deposition, p53-dependent senescence)^53^, and *RRM2* (senescence-associated)^54^ (**Fig. S9a**). GSEA supported enrichment of cellular senescence programs alongside diminished polarity/junctional markers (**Figs. S8** and **S9b**). Morphologically, while AES consistently expanded into 3D structures at earlier passages, beyond P19, they failed to reorganize into sac-like structures and instead remained as compact aggregates of cells (data not shown), further highlighting the onset of senescence. Taken together, our data demonstrates that AES maintains polarity, barrier integrity, and ATII function up to P19, faithfully modeling alveolar morphogenesis and differentiation. However, beyond this point, cultures progressively lose epithelial fidelity, adopting senescent and EMT-like phenotypes. Thus, for mechanotransduction assays and infection studies, P≤18 (specifically P6 to P18, while P1–5 ATIIs were used for expansion and conditioning ATIIs prior to bioprinting) represent the most physiologically-relevant window.

#### 2.4 3D ventilation of AES triggers differentiation via mechanotransduction

**Figure 3:**
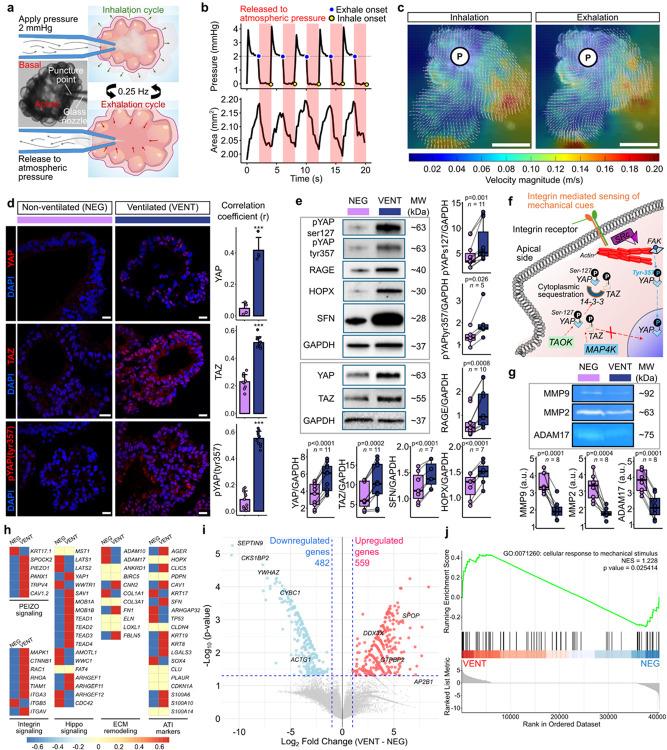
Ventilation induced mechanotransduction in AES triggers ATII-to-ATI transition. a, Schematic illustration of the ventilation mechanism delivering physiologically-relevant cyclic airflow through a punctured glass nozzle for apical actuation of AES. b, Representative ventilation cycle showing pressure oscillations during inhalation and exhalation and corresponding volumetric changes of AES during cyclic actuation. c, PIV-based pixel displacement analysis visualizing global ventilation dynamics, with arrows indicating direction and magnitude of flow-induced motion. White circle labelled ‘P’ marks the puncture site for ventilation delivery. d, Immunostaining of YAP, TAZ, and pYAP (tyr357) showing nuclear translocation following ventilation (VENT 3h+1d; 3h ventilation + 1d static culture), with quantification of nuclear colocalization via correlation coefficient: YAP (*n* = 4, ***p < 0.001), TAZ (*n*= 10, ***p < 0.001), and pYAP (tyr357) (*n* = 11, ***p< 0.001) compared to non-ventilated AES (NEG). e, Representative immunoblots of pYAP (ser127), pYAP (tyr357), RAGE, HOPX, SFN, YAP, and TAZ expression comparing non-ventilated and ventilated (VENT 3h+1d) AES with their densitometric quantification. Blots were representative of multiple independently performed experiments (specified in the figure) with similar results. Non-cropped full blots were included in Fig. S18. f, Schematic depicting integrin-mediated mechanosensing and activation of the Src-FAK pathway, driving nuclear translocation of pYAP (try357) and downstream transcriptional programs. g, Gelatin (for MMP 2/9) and casein (for ADAM17) zymography demonstrating extracellular remodeling following ventilation (VENT 3h+1d) with densitometric quantification. Gels were representative of multiple independently performed experiments (specified in the figure) with similar results. Non-cropped full gels were included in Fig. S18. h, Heatmap illustrating transcriptomic remodeling in non-ventilated and ventilated (VENT 3h+1d) AES. i, Volcano plot of DEGs between NEG and VENT (3h+1d). j, GSEA showing significantly enriched biological pathway associated with ventilation-induced mechanotransduction. Data were presented as mean ± SD, where *p < 0.05, **p < 0.01, and ***p < 0.001.

Cyclic mechanical strain is known to bias ATII fate and epithelial remodeling through mechanotransductive signaling pathways (e.g., Hippo pathways through YAP and TAZ (transcriptional co-activator with PDZ-binding motif))^8,55,56^; but nearly all ex-vivo platforms achieve this with substrate stretch (planar membranes, inverse-opal hydrogels, or chip diaphragms)^16–18,57^. In contrast, we developed a custom-built ventilation actuation platform to deliver air pulses through a microneedle into AES to impose volumetric oscillations of the intraluminal liquid rather than stretching a substrate (Figs. 3a and **S10**). Because AES in Mat are liquid-filled, air did not enter the lumen; instead, pressure transients were transmitted to the apical fluid and the liquid within the microneedle through capillary reaction, driving expansion–contraction of AES in 3D (**Fig. S10a**). This process resulted in the actuation of the apical surface in a cyclic manner. For this study, ventilation was applied at a fixed frequency of 0.25 Hz using custom-developed software (**Fig. S10b** and **Supplementary Software**) and pressure amplitude of 2 mmHg for 3 h, closely simulating the nonstationary dynamics of human tidal breathing^58,59^ (Figs. 3a and 3b). Under the given ventilation, AES responded dynamically to applied ventilation (Fig. 3b and **Supplementary Movie 3**). During the inflation phase (inhalation), AES expanded globally in 3D by up to ~20% compared to their resting state (Fig. 3b). Upon cessation of pressure (released to atmospheric pressure, representing exhalation), AES returned to baseline volume, demonstrating elastic recoil (Fig. 3b). This bidirectional ventilation motion was spatially heterogeneous, persisting even in the farthest region from the punctured needle (Fig. 3c). Ventilation dynamics were initially concentrated near the puncture site (white circle denoted as ‘P’ in Fig. 3c) and propagated peripherally within AES, as expected from the interconnected hollow compartments within the septae, which are analogous to the native alveolar microarchitecture.

To investigate how ventilation alters alveolar epithelial biology, we ventilated AES for 3 h followed by a day of incubation (VENT) and compared them with the negative control (NEG, non-ventilated). Our analysis focused on the Hippo-YAP/TAZ signaling cascade, a known mediator of mechanotransduction and driver of ATII-to-ATI fate transitions^8^. In static conditions (NEG), MST1/2 (STK3/STK4), which are serine/threonine kinases, phosphorylates LATS1/2, which in turn phosphorylates YAP, sequestering it in the cytoplasm and preventing transcriptional activity^60^. Upon mechanical strain^8^ or inhibition of LATS1/2 (e.g., LATS-IN-1)^61,62^, YAP escapes phosphorylation and translocate into the nucleus, where it partners with transcription enhancer activator domain (TEAD) and TAZ to activate gene expression programs critical for ATI differentiation^56,57^. As expected, cyclic ventilation (VENT) induced robust nuclear YAP and TAZ localization compared to NEG, with a significantly higher correlation coefficient indicating nuclear translocation of YAP/TAZ (Fig. 3d). Immunoblotting revealed increased levels of pYAP (Ser127) - a canonical inhibitory phosphorylation that normally sequesters YAP in the cytoplasm (Fig. 3e). To resolve this paradox, we probed for pYAP (tyr357), a phosphorylation site known to promote YAP nuclear translocation^63^. Indeed, both immunostaining and Western blot showed strong induction of pYAP (tyr357) in ventilated AES (Figs. 3d and 3e). Prior studies using 2D cyclic stretch have consistently shown YAP activation through dephosphorylation at Ser127, allowing cytoplasmic YAP to enter the nucleus^64,65^. In contrast, our 3D ventilation system revealed a non-canonical mechanism. While 2D stretch primarily engages basal integrin–cytoskeletal linkages, apical fluid-mediated ventilation in AES activates integrin–FAK (focal adhesion kinase) signaling at the luminal surface, providing a route for Tyr357-dependent YAP activation (Fig. 3f). Functionally, this shift coincided with the induction of alveolar differentiation markers. Ventilated AES displayed higher expression of homeodomain-only protein X (HOPX) and receptor for advanced glycation end products (RAGE) (ATI markers) together with SFN, consistent with the emergence of a PATS/ADI transitional state enroute to ATI differentiation (Fig. 3e). These results indicate that mechanical ventilation accelerates the ATII to ATI transition by engaging non-canonical Hippo inputs. To assess extracellular remodeling post ventilation, we analyzed conditioned media by gelatin and casein zymography. Notably, the reduction in MMP2/MMP9, ADAM17 (a disintegrin and metalloprotease 17) activity under low-amplitude, short-duration ventilation aligns with physiological mechanotransduction (Fig. 3g), where ECM stability supports epithelial polarization and AT2→AT1 progression. This contrasts with non-physiological stretch (e.g., ventilator-induced injury or fibrotic stiffening)^66^, which typically up-regulates MMPs and drives degradative remodeling. This repression may reflect compensatory upregulation of TIMP3 (tissue inhibitor of metalloproteinases 3), which has been reported to accompany ATII to ATI differentiation^67^ and suppress matrix proteolysis. Together, these data suggest that ventilation not only enhances nuclear YAP/TAZ activity via Tyr357 phosphorylation but also promotes the transdifferentiation of ATIIs into ATIs by restraining protease activity in the alveolar niche. To confirm that ventilation-induced remodeling was not accompanied by apoptotic injury, we assessed apoptotic (BAX) and anti-apoptotic (BCL2) markers in samples collected immediately after ventilation (VENT (3h)) and after an additional day of incubation post ventilation (VENT (3h + 1d)) and compared them with NEG (**Fig. S11**). Immunoblot analyses revealed no significant differences between ventilated and non-ventilated controls. These findings indicate that cyclic ventilation did not elicit detectable apoptotic stress, reinforcing the observed epithelial remodeling, ATI/PATS marker induction, and matrix remodeling responses arise from physiological mechanotransducive signaling rather than cell damage. PATS phenotypes were initially described in injury settings and fibrotic remodeling^46^. However, accumulating evidence indicates that KRT8^+^ transitional states (PATS) are not restricted to injury but also appear during physiological differentiation and regeneration^68^. Meanwhile, bulk RNA-seq corroborated that ventilation upregulated multiple components of mechanosensation and force transmission, including *PIEZO1/TRPV4/PANX1/CAV1*^69^ and integrins (*ITGAV/ITGA3*) with Rho-family effectors (*RAC1/RHOA/TIAM1*) (Fig. 3h). Volcano plot of DEGs showed a decrease in *YWHAZ* (14-3-3 protein family), a regulatory adaptor that binds pYAP (Ser127) to retain it in the cytoplasm^70^, a shift that would facilitate nuclear YAP despite persistent Ser127 phosphorylation (Fig. 3i). Moreover, transcriptional changes compatible with YAP–TEAD output and membrane turnover/cytoskeletal pliability, including *DDX3X* (reported co-regulator of transcription), *SPOP* (CUL3 adaptor linked to Hippo components), *AP2B1* (clathrin adaptor for receptor/integrin endocytosis), and reductions in SEPTIN9/ACTG1 (lower cortical constraint) were noted. Finally, GSEA confirmed enrichment of cellular response to mechanical stimulus in ventilated AES (Fig. 3j), aligning the transcriptome with the applied mechanical stimulation.

#### 2.5 Apically delivered influenza infection reveals compartmentalized spread across interconnected AES

**Figure 4:**
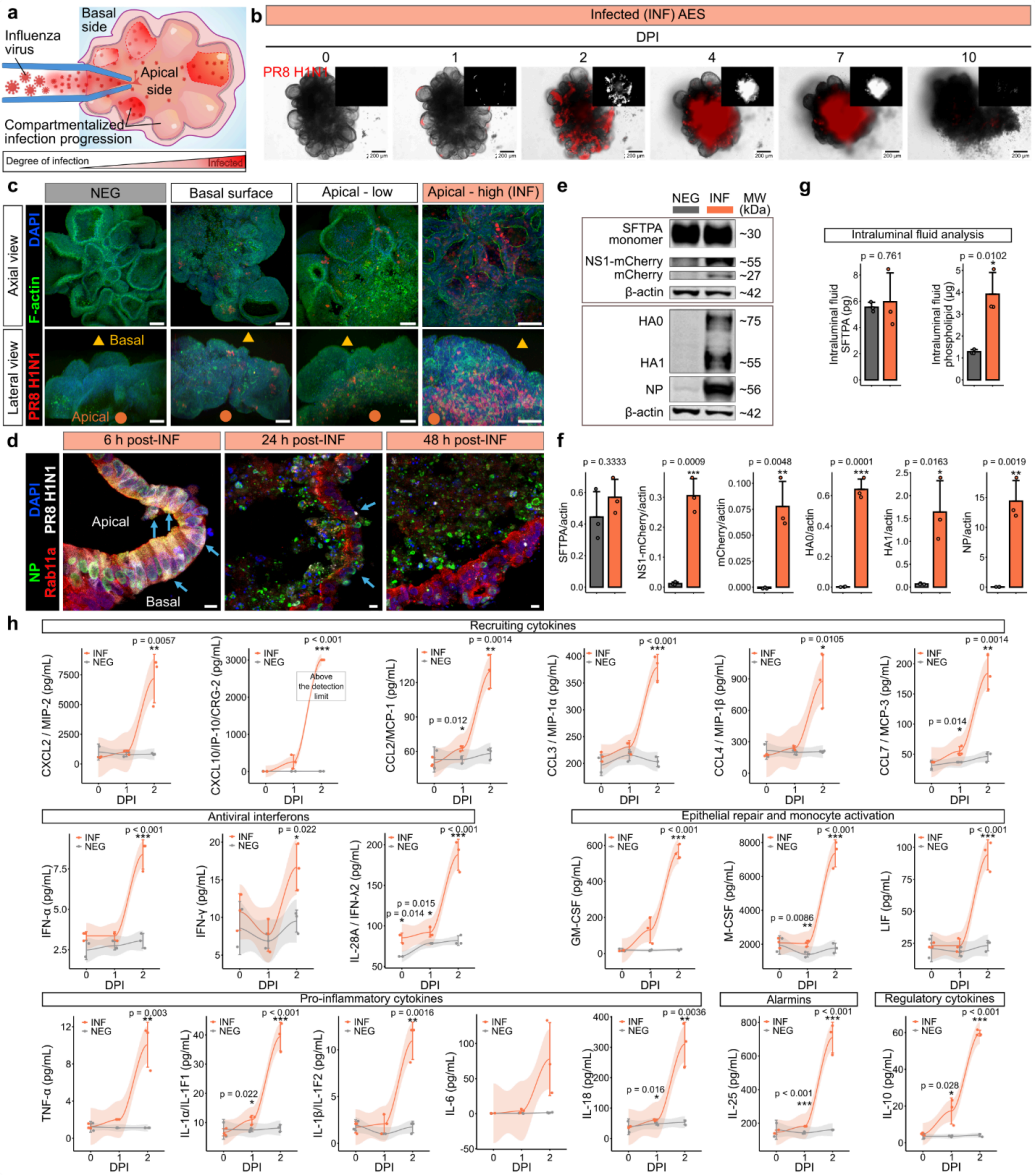
Apical H1N1 infection recapitulates viral propagation and host response in AES. a, Schematic illustration of PR8-H1N1 influenza virus delivery to the apical surface of AES to mimic physiological infection routes. b, Representative time-course images showing propagation of PR8-H1N1 expressing an NS1-mCherry fusion reporter over 10 DPI. Inset shows the mCherry fluorescence channel alone. c, Whole-mount multiphoton images of AES at 10 DPI showing non-infected controls (NEG) and infection outcomes (INF) following basal exposure and apical inoculation with low (apical-low;10,000 pfu) and high (apical-high; 300,000 pfu) viral titers. Scale bar: 100 μm. d, Immunostaining of NP and Rab11a at 2 DPI demonstrating active viral replication and trafficking in AES (blue arrows indicate sites of new viral particle generation). Scale bar: 10 μm. e, Representative immunoblots showing SFTPA, NS1-mCherry, HA, and NP expression in NEG and INF (apical-high) AES. Non-cropped full blots were included in Fig. S20. f, Quantification of protein expression by densitometry normalized to β-actin (*n* = 3 biologically independent replicates). g, Quantitative analysis of SFTPA and phospholipid concentrations in intraluminal fluid from NEG and INF (apical-high) AES at 2 DPI (*n* = 3, biologically independent replicates). h, Temporal profiles of proinflammatory cytokine secretion from NEG and INF (apical-high) AES supernatants at 0, 1, and 2 DPI (0 DPI NEG: *n*= 2; all other groups: *n* = 3 biologically independent replicates). Data were presented as mean ± SD where *p < 0.05, **p < 0.01, and ***p< 0.001.

In native lungs, ATIIs are responsible for host defense against respiratory viruses and for regenerating the alveoli by self-renewal, aiding repair after injury^71^. To model the physiological route of exposure, we delivered influenza A virus (IAV; PR8-H1N1 strain), which expresses an mCherry-tagged nonstructural protein 1 (NS1)^72^, directly into the lumen of AES via microinjection (Fig. 4a and **Supplementary Movie 4**). Unlike Transwell inserts^9–11^ or open-channel lung-chip formats^12–14^, where virions contact an exposed surface or can access the basal side, our AES were closed and liquid-filled; thus, the inoculum was transmitted through the apical luminal fluid of an intact, polarized epithelium. Because each AES comprises ~tens of alveoli, infection evolves compartmentalized within an interconnected cluster, enabling spatially resolved kinetics (Fig. 4b). In our experiments, AES were infected apically at two different viral concentrations: ‘apical-low’ (10,000 plaque-forming units (pfu)) and ‘apical-high’ (300,000 pfu; henceforth referred to as ‘INF’). Control groups included a ‘basal surface’ exposure group (300,000 pfu suspended in medium), which simulated non-physiological viral contact at the basal side, and a punctured control, where PBS was injected to account for any mechanical effects of the microinjection itself. A negative (NEG) control, consisting of non-infected and non-punctured AES, was included. Over 10 days post-infection (DPI), the punctured control retained structural integrity and continued alveolar growth similar to NEG, confirming that the puncture procedure did not disrupt sac morphology (**Fig. S12**). The basal exposure produced minimal mCherry, consistent with preserved apico–basal polarity limiting receptor access. In contrast, the apical-high (INF) group showed early and pronounced alveolar disintegration by 4 DPI, while the apical-low group exhibited a delayed infection trajectory.

Multiphoton imaging at 10 DPI revealed strong NS1-mCherry signal predominantly localized to the apical regions of apically-infected AES, particularly in the apical-high (INF) group (Fig. 4c and **Supplementary Movie 5**), while basal surface group exhibited minimal fluorescence signal. The NS1-mCherry signal was not detected in NEG. Flow cytometry analysis at 10 DPI demonstrated pronounced cell death in infected groups, with live cell percentages of 64.47% and 43.71% in apical-low and apical-high AES, respectively, compared to 88.45% in punctured controls (**Figs. S13a** and **S13b**). Biochemical readouts corroborated productive infection, where CellTiter-Glo 3D viability assays confirmed these results, showing a rapid reduction of metabolic activity within 3 DPI in infected conditions, whereas punctured and negative controls remained stable, affirming minimal impact from the injection procedure (**Fig. S13c**).

To further explore the infection dynamics, high-resolution time courses (6–48 h post infection) revealed early perinuclear NS1, co-localization of nucleoprotein (NP) with Rab11a (supporting viral ribonucleoprotein (vRNP) recycling-endosome trafficking), and apically biased accumulation of NP with loss of MUC1 and focal nuclear fragmentation as barrier failure emerged (Fig. 4d). Importantly, proSPC persisted in a subset of cells, indicating that surfactant-competent ATIIs remain even as infected regions remodel (**Fig. S13d**). Whole-mount NP staining revealed widespread distribution throughout interconnected alveolar compartments, demonstrating native-like interconnectivity and infection propagation patterns in AES (**Fig. S13d**). In addition, INF exhibited a disrupted apical surface as the infection of AES progressed (**Fig. S13e**). In contrast, the NEG group exhibited no detectable NP or mCherry expression, confirming the absence of infection. Immunoblots detected NS1-mCherry, newly synthesized full-length hemagglutinin (HA0) and cleaved subunit HA1, and NP in infected sacs at 2 DPI, with quantification mirroring imaging (Figs. 4e and 4f). SFTPA levels were unchanged between NEG and INF in immunoblots and by analysis of intraluminal fluid (via ELISA), whereas intraluminal phospholipids increased after the infection (Fig. 4g). This may reflect proliferation of viral particles and incorporation of host-derived lipids into viral envelopes, given that detected SFTPA in ELISA did not change within the closed lumen.

Multiplex profiling of supernatants from infected AES revealed a robust, time-dependent cytokine surge (over the 2-day infection period) absent in NEG (Fig. 4h). Recruiting chemokines, including CCL2/MCP-1, CXCL10/IP-10, CCL3/MIP-1α, and CCL4/MIP-1β, were strongly induced, consistent with epithelial-driven recruitment of monocytes, and leucocytes during influenza infection^73–76^, mirroring early chemotactic signals seen in murine influenza bronchoalveolar lavage fluid (BALF) studies^77^. Antiviral interferons, including IFN-α, IFN-γ, and IL-28A (IFN-λ2), also showed a time-dependent increase, reflecting activation of epithelial antiviral responses^76,78,79^. Cytokines associated with epithelial repair and survival were upregulated, including granulocyte-macrophage colony stimulating factor (GM-CSF), which promotes ATII proliferation, alveolar macrophage differentiation, surfactant metabolism, and alveolar macrophage-mediated survival from influenza^80–83^; macrophage colony stimulating factor (M-CSF), which supports alveolar macrophage proliferation; and leukemia inhibitory factor (LIF), which supports epithelial survival. These cytokines indicate a coordinated response to preserve epithelial function^84,85^. Pro-inflammatory cytokines responsible for the acute-phase response and amplification of inflammation, such as TNF-α, IL-1α, IL-1β, IL-6, IL-10, and IL-18, were markedly elevated, demonstrating a robust inflammatory response within infected AES^86^. IL-25, an alarmin released by stressed upper epithelial cells^87,88^, was also significantly increased post infection, although prior evidence for IL-25 production by ATIIs during influenza infection remains limited. Thus, our findings may reflect epithelial stress signaling within closed AES as paracrine inputs akin to airway-like epithelial subsets. IL-10 is known to rise in lung tissue and BALF during influenza infection, largely from immune cells^89^. IL-10 levels increased notably following infection; direct evidence for ATII-intrinsic IL-10 production in influenza remains limited, we hypothesize ATII can secrete IL-10, as part of their regulatory function to communicate with macrophages and help balance the inflammatory response. Upstream (**Fig. S14a**) and canonical (**Fig. S14b**) pathway analysis of bulk RNA-seq data indicate a dominant double stranded RNA (dsRNA) dependent interferon response involving multiple dsRNA sensors. At the same time, feedback control of interferon and IL1b signaling (*IRGM, TREX1, ETV3, RNASEH2B, ILRN*) (**Fig. S14a**) and host mRNA translational processes (**Fig. S14b**) were suppressed, indicating effective control of the host’s translational machinery for expression of viral genes. The graphical summary of the mRNA sequencing data (**Fig. S14c**) shows activation of the ER stress response (EIF2AK3) genes and a central node of IRF7 activation, consistent with occupancy of ER and endosomal compartments by incoming and outgoing virus, respectively. INF showed predominant induction of interferon stimulated genes (**Fig. 14d**). Together, these findings demonstrate that apically delivered virus in a closed, polarized, AES yields a pattern of apical-first entry, polarized trafficking, barrier erosion, interferon inflammation, and compartmentalized spread that models the course of natural infection representing a significant improvement over open-surface or basal-access models that do not recapitulate the alveolar tropism of viral infection.

#### 2.6 Ultrastructural and Transcriptomic Signatures of Differential Susceptibility of AES to Influenza Infection

**Figure 5:**
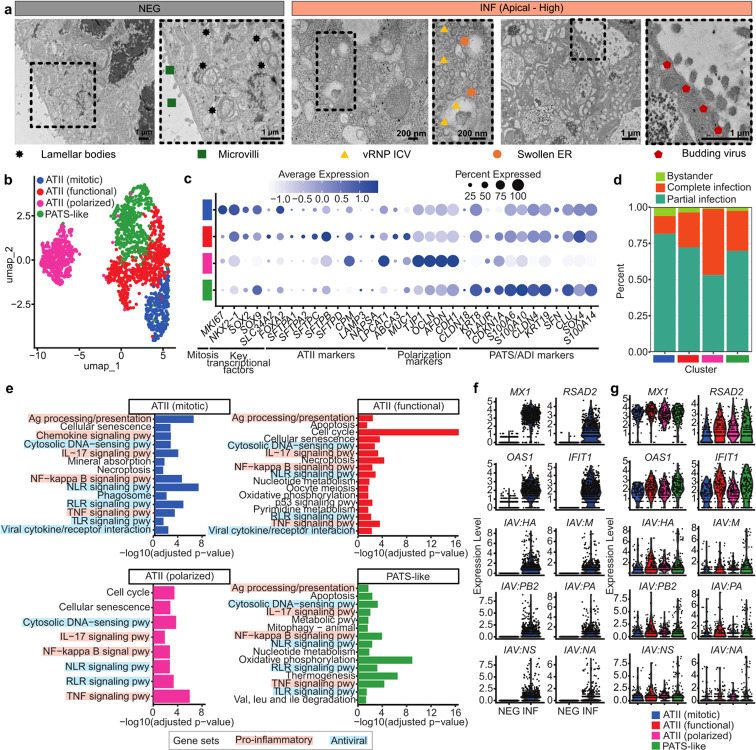
Ultrastructural and single cell profiling of influenza infected AES. a, Representative TEM images of NEG and INF (apical-high) AESs at 2 DPI. Black stars indicate lamellar bodies, green squares denote epithelial microvilli, yellow triangles mark viral ribonucleoprotein (vRNP)-coated irregular vesicles (ICV), orange circles highlight swollen endoplasmic reticulum (ER), and red pentagons indicate influenza budding vesicles. b, UMAP visualization of single-cell transcriptomes from NEG and INF AES at 2 DPI. c, Dot plot showing expression of key gene markers associated with mitosis, transcriptional factors, ATII markers, polarization markers, and PATS/ADI markers in INF AES compared with NEG. d, Proportion of cells classified as bystanders (no viral reads), partially infected (expression of a subset of viral genes), or fully infected (expression of all viral genes) within INF AES. e, Significantly enriched KEGG pathways (adjusted *p* < 0.05) per cluster in INF relative to NEG (adjusted *p* < 0.05 and log_2_ fold change > 0.5). Human diseases and genetic information processing gene sets were excluded from analysis. Pwy = pathway; NLR = Nod-like receptor; RLR = RIG-I-like receptor; TLR = Toll-like receptor; Ag = antigen; Val = valine; Leu = leucine; Ile = isoleucine. f, Violin plots comparing the expression of canonical antiviral ISGs (*MX1*, *RSAD2*, *OAS1*, and *IFIT1*) and viral genes (IAV) between NEG and INF AES. g, Violin plots showing the expression of ISG and viral gene expression across cell clusters in INF AES, highlighting cluster-specific antiviral responses.

To complement cytokine profiling and functional assays, we next examined ultrastructural consequences of apical influenza infection (2 DPI) using transmission electron microscopy (TEM) (Fig. 5a). The NEG group displayed hallmark ATII features including lamellar body-like inclusion and intact epithelial microvilli consistent with preserved surfactant-producing identity. In contrast, the infected AES (INF) displayed cytosolic accumulation of irregular vRNP-coated vesicles (ICVs), adjacent to swollen endoplasmic reticulum (ER), as well as clear budding virions along the apical membrane, supporting active replication and viral assembly. These features mirror canonical descriptions of influenza replication observed within AES to recapitulate native virus–host ultrastructural interactions.

Building upon these cellular and ultrastructural insights, we next applied scRNA-seq to dissect the transcriptional trajectories underlying epithelial remodeling, host defense, and injury responses in infected AES (NEG and INF at 2 DPI). Following integration, we were able to identify four clusters (Fig. 5b): mitotic ATIIs (*MKI67*), functional ATIIs (*SFTPA1*, *SFTPA2*, *SFTPB, SFTPC*, and *SFTPD*), polarized ATIIs expressing junctional proteins (*MUC1*, *TJP1*, *OCLN*, *AFDN*, and *CDH1*), and PATS-like/ADI cells (*KRT8*, *PLAUR*, *CDKN1A*, *S100A6*, *S100A10*, *CLDN4*, *KRT19*, *SFN*, *CLU*, *SOX4*, *S100A14*). Canonical markers confirmed cluster identity, with *SFTPC*, *SFTPB*, and *ABCA3* marking ATII subsets, *CLDN4* and *MUC1* identifying polarized cells, *KRT8*, *SFN*, and *CDKN1A* enriched in the PATS-like population (Fig. 5c). Pertinent to note here is the apparent shift of LPCAT1 expression from ATIIs (functional) in Fig 2h–I to ATIIs (polarized) in Fig 5c is due to the differences in the underlying embedding and scaling strategy; the former was generated from baseline data alone whereas the latter reflects a new embedding of both NEG and INF at 2 DPI. The internal scaling of dot plots within the INF subset highlights the polarized ATII population as the highest LPCAT1-expressing group in that specific context. The ATII-polarized cluster showed lower surfactant transcript abundance despite retaining ATII identity, likely representing transitional ATIIs undergoing apical maturation, consistent with prior reports that polarity acquisition precedes full surfactant maturation^41^ and accompanied with dependence on *LPCAT1*-mediated phosphatidylcholine remodeling^90^. The INF samples showed significant changes in immune response, repair, and surfactant dysfunction in response to viral infection compared to NEG. Stratifying by infection state revealed clear differences in viral burden: polarized ATIIs and PATS-like cells exhibited higher proportions of complete infection (~45 and ~27% respectively), while mitotic and functional ATII populations exhibited ~12 and ~24%, respectively (Fig. 5d). This suggests that despite evidence of cell cycle activation within the functional ATII population (Fig. 5c), these cells remain more permissive to viral replication than the actively dividing mitotic ATII subset. This was corroborated by pathway enrichment studies, which indicated these following observations (Fig. 5e). Mitotic ATIIs upregulated DNA-sensing, NF-κB, and TNF signaling pathways, consistent with stress and inflammasome activation. Functional ATIIs exhibited broad enrichment in antigen presentation, phagosome activity, and apoptotic signaling, aligning with their role as active antiviral responders. All clusters exhibited activation of IL-17 and NF-κB signaling pathways, along with antiviral pathways such as NLR, RLR, TNF, positioning them as key compartments in the antiviral response. Despite the strong antiviral response as a result of acute IAV infection, epithelial cell subsets support viral gene expression at both mRNA (Figs. 5f and **5g**) and protein levels: *HA*, *NA*, PB2, and *NEP* transcripts were readily detected in all the subsets (Fig. 5g) consistent with production of viral progeny (Fig. 5a). Host antiviral interferon-stimulated genes (ISGs): *MX1*, *RSAD2*, *OAS1*, *IFIT1*, were induced across all infected clusters, but *OAS1*, *IFIT1* were higher in polarized ATII and PATS-like populations, mirroring their viral transcript burden. Together, these data reveal that viral infection was not uniformly distributed across alveolar epithelial states. Instead, proliferating ATIIs were either resistant to viral infection or better at eradicating the virus, whereas functional and polarized ATIIs, and PATS-like intermediates, sustain high viral replication burden and mount strong interferon responses. This compartmentalization reflects the native susceptibility gradient of alveolar epithelium and highlights how our model recapitulates physiologically-relevant infection patterns.

## Discussion

During embryonic and postnatal lung development, alveologenesis is critically dependent on epithelial-mesenchymal crosstalk. The mesoderm component guides the canalicular and saccular stages of alveologenesis. Importantly, mesodermal derivatives, including PDGFRα^+^ fibroblasts and lipofibroblasts, provide essential niche signals such as FGF-10 (fibroblast growth factor-10) that promote distal epithelial outgrowth, while vascular and immune components further shape alveolar maturation and homeostasis^3^. In line with this, in-vitro assays historically required mesenchymal feeders to sustain clonal expansion of ATIIs (murine) and to support their differentiation into ATIs, as exemplified by the ‘alveolosphere’ culture described by Barkauskas et al.^91^. Pertinent to note here, mesenchymal support invariably has challenges due to niche complexity and signal variability from different cells that constitute the niche. To overcome this, one ideal way forward can be to adopt mesenchyme-free, epithelial-only alveolosphere-based systems in part to eliminate inter-batch variability and confounding secretome heterogeneity inherent in stromal fibroblast cultures, relying instead on precisely tuned, defined media to supply key developmental cues (Wnt/FGF/BMP/glucocorticoid/cAMP). Subsequent studies^15,62,92,93^ extended this paradigm by showing that iPSC-derived NKX2–1^+^ progenitors can generate ‘epithelial-only’ ATIIs and ATI-based alveolospheres that exhibit surfactant processing and lamellar body formation. The ability of these iPSC-derived ATIIs to form alveolospheres is due to the presence of lineage-coded circuits for polarity, lumenization, and surfactant biogenesis within these epithelial cells. This process is facilitated by staged cues (Wnt agonism via CHIR, followed by FGF supplementation in the defined media). Thus, culturing iPSC-derived ATIIs in this defined media unlocks these epithelial programs, where NKX2–1^+^ progenitors rapidly acquire ATII identity, assemble into lumen containing alveolospheres, and mount innate immune responses, all without mesenchymal feeders.

While adopting the protocol pertaining to iPSCs-derived ATII-derived alveolospheres in our cultures, we noticed bundled alveolospheres in regions of high local cell density, suggesting that aggregate packing promotes alveolar morphogenesis. However, a former study note that plating these ATIIs during passaging too densely (>400–1000 cells/μl) hindered clonality and reduced differentiation efficiency^15^. Therefore, the observation we noticed was due to stochastic random aggregation and not by deterministic self-organization. Our approach deliberately embraces high-density conditions but does so in a geometrically confined manner, akin to ‘bioprinting-assisted tissue emergence’ (BATE) concept^94^. In this context, high cell density is not a detriment but rather a driver for controlled fusion and remodeling, enabling reproducible sac-like architectures to obtain AES. AES adds higher-order geometry harnessing the deterministic self-organization, which yields interconnected polarized epithelial sheets that lumenize into multi-unit, alveoli-form assemblies. Mechanistically, this scales alveolosphere-based system’s self-organization from single cysts to bundled sacs, which we describe is driven through integrin-driven polarity and RAC/PAK remodeling. Moreover, single-cell transcriptomics reveal AES recapitulating the expected heterogeneity of the alveolar epithelium, comprising proliferative ATIIs, functional surfactant-secreting ATIIs, and PATS/ADI-like transitional states. The PCP alignment orchestrates budding and compartmentalization across the sheet and also exhibits ATII plasticity to acquire PATS/ADI phenotype^42^ as AES grow and mature (14 DPI). While true septation *in vivo* requires stromal elastogenesis and vascular scaffolding^3^, our mesenchyme-free AES establish closed, barrier-competent epithelia with apical access enabling 3D ventilation and physiologically-relevant apical infection studies that surpass single-cyst organoids, positioning it at a higher in structural hierarchy than alveolospheres. Moreover, epithelial-only AES platform isolates intrinsic morphogenetic programs of alveolar epithelium and is helpful in delineating the response of epithelial compartment in host defense.

Another advancement in our study is the ventilation model that it fundamentally different from prior “stretch-only” models by ventilating a closed, liquid-filled epithelium, where pressure pulses drive whole-sac volumetric oscillations transmitted through the apical fluid, not just 2D substrate strain^16–18^. This 3D actuation produces spatially propagated deformation across interconnected compartments, i.e., a mechanics regime closer to native alveolar filling. In addition, ventilation forces are not distributed uniformly across sacs; some regions experience greater actuation while others remain under stimulated, resulting in mechanical heterogeneity similar to that observed *in vivo*. In this context we observe non-canonical Hippo engagement—robust YAP/TAZ nuclear entry accompanied by tyr357 phosphorylation—consistent with apical integrin–FAK inputs in addition to the classic ser127 dephosphorylation seen on basal stretch. To the best of our knowledge, non-canonical YAP activation via tyr357 phosphorylation in alveolar epithelium under mechanical strain has not been directly demonstrated. Yet, given that integrin–FAK and Src pathways can phosphorylate YAP at tyr357 in other epithelial contexts^95,96^, our observation of tyr357-driven YAP nuclearization in ventilated AES likely reflect a physiologically-relevant mechanotransductive axis that has simply not been closely examined *in vivo*. Howbeit, our mechanics-first strategy avoids genetic manipulation and chronic kinase inhibition^62^, elicits AT1-like transitions within an intact, polarized sac, and naturally couples differentiation with barrier stabilization and protease restraint: features that are difficult to recapitulate with uniform small-molecule activation alone^61,62^. A balanced view is that chemical YAP/AKT modulation offers scalable, tunable positive controls for AT1 induction^61,62^, whereas 3D ventilation exposes how epithelial mechanosensing organizes that fate choice in space and time. Despite the strengths in the ventilation approach, several limitations persist. The current ventilation setup is not compatible with long-term (e.g., a week of ventilation) ventilation, primarily due to technical barriers including potential leakage at the puncture interface from the transfer to the incubator, needle clogging from intraluminal cell debris, manual intervention and the absence of automated feedback to maintain consistent pressure. Moreover, while ATII-to-ATI transitions were initiated, terminal differentiation into mature ATI remains incomplete, likely due to the absence of key microenvironmental signals from endothelial, immune, and stromal populations. Future iteration can benefit from inclusion of such microenvironmental cues and future integration of multi-port actuation or closed-loop pressure feedback may help overcome these limitations.

A central advantage of our model is the ability to study respiratory viral infection in a closed, apically accessible epithelial sac. Unlike Transwell or chip systems that expose basal or open apical surfaces, microinjection into AES containing ~40 alveoli achieve physiological apical entry routes and compartmentalized infection dynamics recapitulating canonical antiviral signaling responses. Despite identical viral load (300,000 pfu) to the basal surface group, the basal inoculation of alveolar sac failed to demonstrate robust infection, likely due to the limited virus penetration through Mat and inefficient viral access to apical entry receptors through the tight lateral barrier. PR8-H1N1 utilizes sialic acid-bearing receptors, which are predominantly expressed on the apical surface of alveolar epithelium^97,98^. As expected, apical infection led to significantly stronger ISG responses, verifying the maintenance of apical-basal polarity and confirming that viral entry occurred in a physiologically-accurate manner. Additionally, viral infection induced transcriptomic changes mimicking injury-induced alveolar repair. Infection led to downregulation of surfactant genes (*SFTPA1*, *SFTPC*, *SFTPD*), cellular transition from functional ATIIs to proliferative ATIIs (mitotic), and PATS-like states, mimicking injury-induced alveolar regeneration pathways overserved *in vivo*. The expansion of ATII (mitotic) clusters and the depletion of surfactant producing ATII (functional) populations collectively suggest that the model replicates the early reparative transition triggered by viral injury, in line with in-vivo regenerative responses. The ATII (functional) cluster in INF exhibited upregulation of cell cycle–associated genes (Fig. 5c), indicating that a substantial fraction of these cells turned into proliferative state after infection. This proliferative state may facilitate progressive viral spread across the epithelial layer over time, as cells at distinct stages of the cell cycle display differential susceptibility to lytic infection. Notably, cells in mitosis appeared less permissive to infection (Fig. 5d), likely due to sequestration of microtubule machinery for mitotic spindle assembly, thereby limiting its availability for endosomal trafficking and subsequent viral replication, export, packaging, and release of viral progeny. This capacity to transition from surfactant-secreting to proliferative phenotypes marks a critical advantage of the model in studying epithelial plasticity under pathogenic stress, where induction of high levels of GM-CSF may counteract the anti-proliferative effects of interferons in an autocrine-dependent manner. In addition, taking advantage of closed-AES, we were able to collect intraluminal fluid to characterize SFTPA and phospholipid. The significant elevation of choline-containing phospholipids in the intraluminal fluid following infection, in contrast to the unchanged SFTPA protein levels (Fig. 4g), provides mechanistic insight into host–virus interactions within AES. This lipid enrichment is consistent with the well-established composition of influenza virions, which acquire their envelope from the host plasma membrane, incorporating substantial amounts of phosphatidylcholine and sphingomyelin during budding^99^. Such changes likely reflect increased membrane turnover and lipid remodeling associated with viral replication rather than enhanced surfactant synthesis by ATIIs. Moreover, the lack of detectable differences in total SFTPA by ELISA, despite transcriptional evidence for SFTPA1 and SFTPA2 expression and proteomic identification of SFTPA, suggests possible post-transcriptional regulation, differential degradation, or intracellular accumulation of surfactant proteins during ER remodeling and export of the viral genome segments to budding virions. Pertinent to note here, influenza virions are enriched in sphingomyelin and phosphatidylcholine and utilize cholesterol and sphingomyelin-rich lipid rafts for budding^99^, so the observed phospholipid increase may also signify enhanced raft-associated membrane remodeling during virion formation. Future studies using lipidomics with higher resolution or isoform-specific surfactant assays may help clarify the relationship between lipid remodeling, surfactant homeostasis, and viral replication dynamics. In addition, lipid speciation (using mass spectrometry) and normalization to viral protein (NP/HA) in future experiments will help disentangle host surfactants from virion-associated lipids. Transcriptomic and proteomic analyses suggest that an imbalance in sensing of endogenous versus viral dsRNA may bias the innate response towards inflammation and injury rather than effective control of the infection. Together, future iterations in AES can incorporate alveolar macrophages and vascular networks, which can improve endothelial maturation and enable modeling of more complex cell-to-cell and immune-epithelial interactions. Integration of vascularized compartments, as recently described in organoid vascularization studies^100^, could address this limitation and advance the model toward more complete alveolar mimicry.

ECM environment is another critical factor. We used Mat, which, although widely employed in organoid research, suffers from batch-to-batch variability. Although we observed consistent sac formation across Mat batches, synthetic or decellularized ECM scaffolds with tunable mechanical and biochemical properties may offer improved control over tissue architecture, epithelial polarity, differentiation, and alveolar function. Although the apical surface is accessible for infection and sampling, another limitation is the inability to establish a complete air-liquid interface in 3D, which restricts remodeling of gas exchange and surfactant dynamics over long-term culture. Achieving a true 3D air–liquid interface remains an unmet goal, but advances in engineered models and perfusion strategies may bring this within reach.

In summary, our AES platform represents a mesenchyme-free alveolar model that extends the classical alveolosphere paradigm into higher-order, closed sac-like assemblies with apical access, mechanosensitive remodeling, and infection competence. While limitations remain such as incomplete ATII-to-ATI maturation, and absence of vascular or immune niches; the system faithfully captures core epithelial programs of polarity, surfactant biology, plasticity, and mechanotransduction. Together, AES provide a tractable framework to dissect intrinsic alveolar epithelial dynamics and offers a foundation for future integration of stromal, vascular, and immune components toward full alveolar mimicry, while showcasing its potential to transform in-vitro modeling of lung diseases, regenerative responses, and antiviral therapies.

## Materials and Methods

### Generation of ATIIs from iPSCs

ATIIs were generated from iPSCs (BU3 line from the Center for Regenerative Medicine at Boston University) following a recent study^15^. Briefly, iPSCs were cultured on Mat-coated 6-well plates under feeder-free conditions for 3–4 passages in mTeSR Plus medium (STEMCELL technologies, 100–02756) before ATII differentiation. iPSCs were first differentiated into definitive endoderm cells using the definitive endoderm kit (STEMCELL technologies, 05110) for 3 days. Next, the definitive endoderm was further differentiated into anterior foregut endoderm with dorsomorphin (DS; 2 μM, STEMCELL technologies, 72102) and SB431542 (SB; 10 μM, Tocris, 1614), inhibitors of bone morphogenetic protein (BMP) and transforming growth factor (TGF)-β, respectively, which were supplemented to complete serum-free differentiation medium (CSFDM). This medium, referred to as (DS/SB), was used for 3 days. CSFDM medium consisted of a 3:1 ratio of IMDM (Iscove’s Modified Dulbecco’s Medium)/Ham’s F12 supplemented with Glutamax (1:100, Thermo Fisher Scientific, 35050061), N-2 (0.5X, Thermo Fisher Scientific, 17502–048) and B-27 (0.5X, Thermo Fisher Scientific, 17504–044), bovine serum albumin (BSA; Sigma, A9418), ascorbic acid (50 μg/mL, Sigma, A4544), and 1-thioglycerol (4.5 × 10^−4^ M, Sigma, M6145), and primocin (100 ng/mL, Invivogen, ant-pm-1). To induce differentiation into NKX2–1^+^ lung progenitors, the anterior foregut endoderm was further activated with CHIR99021 (Wnt pathway activator) (C) (3 μM, Cayman Chemical, 13122), recombinant BMP-4 (B) (10 ng/mL, PeproTech, 120–05), and retinoic acid (Ra) (100 nM, Sigma, R2625) in CSFDM media (CBRa). Lung progenitors were then sorted based on CD47^hi^/CD26^lo^ expression to enrich for NKX2–1^+^ lung progenitors, and the sorted cells were confirmed through intracellular staining for NKX2–1 expression. NKX2–1^+^ cells were embedded in Mat (Corning, 356231) at a density of 400 cells/μL and cultured in the CKDCI medium supplemented with CHIR99021 (3 μM), recombinant human keratinocyte growth factor (KGF; 10 ng/mL, PeproTech, 100–19), dexamethasone (50 nM, Sigma, D4902), 8-Bromoadenosine 3′,5′-cyclic monophosphate sodium salt (1mM, Sigma, B7880), and 3-Isobutyl-1-methylxanthine (1 mM, Sigma, I5879) in the CSFDM medium. Over time, embedded lung progenitors differentiated into SFTPC^+^ ATIIs. These ATIIs were maintained in the CKDCI medium and Mat, with medium changes every 2 days and passaging performed every 14 days to ensure maturation and continued growth. For passaging, Mat-embedded alveolospheres were enzymatically released using Dispase II (2 mg/mL, Thermo Fisher Scientific, 17105041), disassociated into single cells with trypsin-EDTA (Corning, 25–051-CI), and re-embedded in fresh Mat at a concentration of 400 cells/μL. 50 μL Mat droplets were placed centrally in each well of a 12-well plate and allowed to solidify at 37 °C for 30 min before adding the CKDCI medium. All experiments in this study were conducted using ATIIs between passages 6 and 18 (P6–18), except for those shown in **Figs. S8** and **S9**, which utilized later passage to access senescence onset.

### Flow cytometry and cell sorting

To obtain single-cell suspensions for flow cytometry and fluorescence-activated cell sorting (FACS) during ATII differentiation, 1 mL of gentle cell dissociation reagent (STEMCELL technologies, 100–0485) was added to each well of a 6-well plate and incubated at 37 °C for 8–10 min. Following detachment, cells were diluted with an equal volume of Dulbecco’s modified eagle medium (DMEM)/F12 (Cytiva, SH30126.FS) containing 10 μM Y-27632 (Tocris, 1254) and gently dissociated by pipetting twice using a P1000 pipette. The cell suspension was centrifuged at 200 × g for 5 min, the supernatant was discarded, and the cell pellet was resuspended in FACS buffer composed of phosphate-buffered saline (PBS; Corning, 21–031-CV), 2% FBS (R&D Systems, S11150) and 10 μM Y-27632. Cells were passed through a 40-μm cell strainer (Corning, 352340) to remove aggregates and ensure single-cell preparation. Cells were then stained with primary antibodies for 30 min on ice. For intracellular staining, cells were first fixed in 1.6% paraformaldehyde (PFA; Santa Cruz Biotechnology, sc-281692) at 37 °C for 10 min, permeabilized using an intracellular permeabilization wash buffer (BioLegend, 421002) at room temperature for 30 min prior to primary staining. After staining, samples were centrifuged at 300 × g for 5 min and washed twice with FACS buffer. All antibodies and their concentrations used in this study are listed in **Supplementary Table 1**. To assess cell viability in non-bioprinted, bioprinted, and virally infected samples, Calcein Violet AM (Invitrogen, C34858) was added at a dilution of 1:1000. Flow cytometry was performed using BD LSR-Fortessa, and cell sorting was conducted using a Bigfoot cell sorter (Thermo Fisher). Data were analyzed using accompanying software (BD, FACSDiva^™^) and Kaluza software (Beckman Coulter).

### Bioprinting of AES

For bioprinting of ATIIs, alveolospheres cultured in Mat were collected by digesting Mat with 2 mg/mL of Dispase II solution. The collected alveolospheres were trypsinized and centrifuged to isolate cell clusters. After centrifugation, the supernatant was completely discarded to obtain cell pellets, as residual medium can affect bioprinting results due to gradients in cell distribution within the nozzle. These pellets were gently loaded into a pulled glass pipette with a final inner diameter of 100 μm, which was connected to a gas-tight 25 μL syringe (Hamilton, 1702) pre-filled with the CKDCI medium. The inner diameter of the glass nozzle did not affect the controllability of patterned cell clusters, as volumetric injection was independent of the nozzle diameter. The cell-loaded syringe was carefully mounted onto a vertically positioned syringe pump integrated within the bioprinting platform. This platform was equipped with a high-precision XYZ linear stage for 3D movement^101^, a bottom camera to monitor the bioprinting process in real time, and the syringe pump to accurately control the volume of cell pellet deposition.

A 3D-printed polylactic acid device (acting as mold for bioprinting, Fig. 1a and **Supplementary file 1**) was designed for patterning ATIIs in Mat. A cover glass was attached to the bottom of this device using polydimethylsiloxane (PDMS), enabling visualization of the bioprinting process via the bottom-view camera. The 3D-printed devices were cured at 37 °C overnight, followed by immersion in 70% ethanol and air-drying in a biosafety cabinet overnight with ultraviolet exposure for sterilization.

For the bioprinting process, a 55-μL mixture of Mat and CKDCI medium in a 1:1 ratio was loaded onto the device inside the bioprinting setup and incubated for 1 min at 37 °C prior to bioprinting. The cell-loaded glass nozzle was placed inside Mat using motion control and deposition was performed using the syringe pump, which facilitated precise control of cell density during bioprinting. Each device contained three ATII clusters (Fig. 1a). ATII pellets were injected at defined injection volumes (e.g., 0.01, 0.05, and 0.1 μL) to modulate cell density during bioprinting. Bioprinted devices were incubated for 30 min at 37 °C to allow Mat to fully solidify, followed by the addition of 200 μL of the CKDCI medium supplemented with 10 μM of Y-27632 (for the initial 2 days of culture) to each device. Bioprinted ATIIs were then cultured in a humidified CO_2_ incubator at 37 °C, with the CKDCI medium replaced every 2–3 days.

To evaluate the controllability of cell density during bioprinting, various volumes (0.001, 0.005, 0.01, 0.05, 0.1, 0.2, and 0.5 μL) of ATIIs were injected and collected for analysis. DNA and protein content in samples were measured using Quant-iT^™^ PicoGreen^™^ dsDNA Assay Kits (Thermo Fisher Scientific, P7589) and Pierce^™^ BCA Protein Assay Kits (Thermo Fisher Scientific, 23227), respectively. The assays were performed according to the manufacturer’s instructions.

To investigate the effect of deposited cell clusters on AES formation, different cell densities were deposited at different injection volumes (e.g., 0.01, 0.05, and 0.1 μL), and the growth of AESs was observed using a Zeiss Axio Observer microscope. To quantify the 3D volume of the formed AESs on 20 DPB, a Zeiss Lightsheet 7 microscope was used after staining with Alexa Fluor 488 (Cayman Chemical, 20549) at a concentration of 1:200. Scanned images were reconstructed into 3D models, and the 3D models were converted into an STL (stereolithography) format. The solid volume of AESs was then calculated using Blender software.

For the evaluation of epithelium barrier integrity, 4 kDa FITC-labeled Dextran solution (2 mg/mL, diluted in the DMEM medium without phenol red, Sigma-Aldrich, 46944) was utilized following a published study^102^. Briefly, the developed live AES were collected and resuspended into separate tubes containing normal sample (CKDCI medium only) and positive control (2 mM EDTA in PBS) on ice for 15 min. The EDTA-treated AES were gently washed with the CKDCI medium three times to remove the EDTA residue. Samples were resuspended in the 4 kDa FITC-Dextran solution (2 mg/mL). Samples were then gently mounted on a glass microscope slide marked with a circle by a hydrophobic circle using a peroxidase-antiperoxidase pen. A coverslip was gently placed on top of the spot marked with vacuum grease on the slide to immobilize the samples. FITC was detected together with differential interference contrast using the Zeiss LSM 880 confocal microscope. The obtained images were analyzed for the percentage of FITC intensity permeabilized inside the samples using ImageJ software.

### Ventilation of AES

ATII clusters were bioprinted into a device with dimensions of 5 mm × 5 mm × 2 mm (**Supplementary file 2**). Following formation (~14 DPB), AES were utilized for ventilation. To facilitate intraluminal ventilation, a ~50 μm-wide glass nozzle tip was inserted through a 3D-printed lid (**Supplementary file 3**) and positioned to align with the center of each AES. The lid and puncture nozzle were immobilized using an adhesive following successful confirmation of the puncture. The glass pipette was connected to the ventilation platform, and ventilation was monitored in real time using a camera (**Fig. S10a**). In parallel, non-ventilated AESs were maintained under static atmospheric pressure as controls. AESs were ventilated at a frequency of 0.25 Hz by alternating between a positive pressure of 2 mmHg (inhalation phase) and atmospheric pressure (exhalation phase) for a total duration of 3 h at room temperature in the biosafety cabinet. Ventilation was controlled via custom-built software interface (**Fig. S10b** and **Supplementary Software**) and a custom-designed ventilation platform incorporating solenoid valves for digital air pressure regulation. The ventilation-induced motion of AES was recorded and displayed in real time on a video during ventilation. Sequential frames were extracted from the video and imported into an open-source MATLAB-based toolbox PIVLab for PIV (particle image velocimetry) analysis. The analysis was performed to quantify pixel displacement and calculate velocity magnitude across the field of view. The resulting vector fields were used to visualize and quantify global deformation and airflow dynamics within AES. Velocity magnitude maps were generated to depict the direction and intensity of tissue motion during ventilation. After ventilation, both ventilated and non-ventilated AES were cultured for an additional static condition in the incubator prior to collection for immunostaining, Western blotting, and RNA seq.

### Viral infection

For influenza A infection, mCherry^+^ A/Puerto Rico/8/1934 influenza virus (referred to “PR8-H1N1” or “PR8-H1N1-mCherry”, Schotsaert Lab, Mount Sinai^72^) was used. PR8-H1N1 (6 × 10^8^ pfu/mL, stock concentration) was suspended in the RPMI 1640 medium containing 5% FBS to prepare a final concentration of 1 × 10^8^ pfu/mL. Viral particles were loaded into a pulled glass micropipette (20-μm diameter) and injected into the central region of each AES using a syringe pump, ensuring exposure to the apical side of the epithelium. Puncturing was performed with a motorized stage and a brightfield microscope (Motic, BA310). For the apical-low group, 0.1 μL (10,000 pfu) of virus-suspended RPMI medium was injected, while for the apical-high group, 3 μL (300,000 pfu) was injected on the apical surface. For the basal surface group, 3 μL (300,000 pfu) of virus-suspended RPMI medium was added to the CKDCI media without puncture or injection. As a non-infected control for the punctured group, 3 μL of PBS (without viral particles) was injected into the apical side of AES. To evaluate the effect of puncture-induced damage, a non-punctured, non-infected group was included as a negative control.

Morphological changes in infected AES were observed using the Zeiss Axio Observer microscope over 10 DPI, using both brightfield and mCherry fluorescence channels. To quantify fluorescence intensity post infection, the mean mCherry fluorescence intensity was measured and normalized to the initial (pre-infection) intensity for each group using ImageJ software.

To assess cell viability post infection, a CellTiter-Glo 3D cell viability assay kit (Promega, G9681) was used. Samples from each experimental group (negative, punctured, basal surface, apical-low, and apical high) were collected at 1, 2, and 3 DPI and washed with PBS. The assay was followed according to the manufacturer’s instructions. Briefly, the CellTiter-Glo 3D reagent was added in an equal volume to the cell culture medium, incubated at room temperature for 30 min, and luminescence was measured using a microplate reader (Tecan, Infinite 200 Pro). Luminescence values at 1 DPI were normalized to the respective group values.

### Multiplex cytokine assay

For proinflammatory response analysis, 150 μL of supernatants from both NEG and INF samples were collected every 24 h (0, 1, and 2 DPI) and immediately replaced with an equal volume of fresh CKDCI media. Collected supernatants were stored at −80 °C until analysis. Cytokine and chemokine levels were quantified using a human magnetic Luminex assay kit (R&D systems, LXSAHM) for multiplexed analysis. The assay was performed according to the manufacturer’s instructions to measure the concentrations of secreted cytokines and chemokines.

### Intraluminal fluid analysis

Intraluminal fluid from both NEG and INF bioprinted AESs was collected at 2 DPI. Fluid extraction was performed using a syringe pump in a pulling mode following micropipette puncture, using the same microinjection setup applied during viral infection. Collected samples were stored at −80 °C until further analysis. For SFTPA detection, a human SFTPA ELISA kit (Novus Biologicals, NBP2–76692) was used, following the manufacturer’s instructions.

The intraluminal fluid samples were used to measure phospholipid content. A colorimetric phospholipid quantification assay kit (Sigma-Aldrich, CS0001) was used following the manufacturer’s instructions.

### Electron microscopy

Field emission scanning electron microscopy (SEM, Apreo, Thermo Fisher Scientific) was used to investigate the pores of Kohns in AES. Bioprinted AES were harvested from the Mat-embedded device using Dispase II solution (2 mg/mL) for 1.5 h at 37 °C. The harvested AES were fixed in 4% PFA for 1 h, followed by washing in PBS (1X) to remove the fixative. The samples were then dehydrated using graded ethanol concentrations (25, 50, 70, 80, 90, and 100%). To maintain the structural morphology of AES, samples were further dried using a critical point dryer (CPD300, Leica) for 4 h. After dehydration, the samples were sputter coated with iridium using a sputter coater (Leica) and observed using SEM.

For transmission electron microscopy (TEM), samples were harvested similarly to those for SEM. The collected samples were fixed by 2.5% glutaraldehyde and 2% PFA in 0.1 M phosphate buffer (pH 7.4) and further fixed in 1% osmium tetroxide in 0.1 M phosphate buffer (pH 7.4) for 1 h. They were then dehydrated through a graded ethanol series, treated with acetone, and embedded in LX-112 (Ladd Research). Ultrathin sections (65nm) were stained with uranyl acetate and lead citrate and examined using a JEOL JEM1400 TEM (JEOL USA Inc.) located at the Penn State College of Medicine TEM Facility (RRID Number: SCR_021200).

### Western blot analysis

Collected AES were lysed in 60 μL of SDS Lysis buffer (1% sodium dodecyl sulfate, 50 mM Tris(hydroxymethyl)aminomethane hydrochloride pH 8.1, 10 mM EDTA pH 8.0) supplemented with 1× Protease/Phosphatase Inhibitor Cocktail (Cell Signaling Technologies, 5872S) and 1× LDS sample buffer (Invitrogen, NP007). Samples were thawed, sonicated at 50% amplitude for 10 sec in 2 sec ON/OFF intervals, incubated at 95 °C for 5 min, and vortexed.

For development and ventilation related studies, proteins were separated on 4–12% 4–20% Mini-PROTEAN^®^ TGX^™^ (Tris-Glycine eXtended) Gels (Bio-Rad, 4561094) and transferred using the Mini-PROTEAN^®^ Tetra electrophoresis wet transfer system (Bio-Rad, 1658033FC) onto an Immun-Blot PVDF Membrane (Bio-Rad, 1620177). Blots were blocked in 5% nonfat dry milk (Bio-Rad, 1706404) made in Tris-buffered saline containing 0.1% Tween 20 (TBST). Blots were incubated overnight at 4 °C with primary antibodies (listed in **Supplementary Table 1**) diluted in 1X TBST (from 10X TBST, Cell Signaling Technology, 9997S) containing 2% nonfat dry milk. Following primary incubation, blots were washed and incubated for 2 h at room temperature with HRP-conjugated secondary antibodies (listed in **Supplementary Table 1**) diluted in TBST with 2% nonfat dry milk. Blots were imaged using the Bio-Rad ChemiDoc MP Imaging System. Prestained ladder (10 – 245 kDa) (Abcam, ab116028), Precision Plus Protein^™^ WesternC^™^ Blotting Standards, 10–250 kDa (Bio-Rad, 1610376), Precision Protein^™^ StrepTactin-HRP Conjugate (dilution:1:10,000), Bio-Rad 1610381) were used as reference markers for determining the molecular weights of proteins of interest. When required, blots were stripped using Restore^™^ PLUS Western Blot Stripping Buffer (Thermo Scientific, 46428) according to manufacturer’s instructions, thoroughly washed in TBST, and then reprobed with additional primary antibodies. After acquisition, densitometric analysis was performed using Image Lab (Bio-Rad, v5.1, build 8). Image brightness and contrast were uniformly adjusted in Image Lab. Band intensities were quantified after local background subtraction done through Image Lab and normalized to GAPDH loading controls. Only linear-range exposures were used for quantification, and adjustments to image brightness/contrast were applied uniformly across the entire blot without altering quantitative values. Normalized intensities were exported from Image Lab and were reported as fold-change relative to the indicated control.

For infection studies, proteins were separated on 4–12% Bis-Tris gels (Invitrogen, NP0321BOX, NP0349BOX) and transferred using the eBlot L1 Transfer system (GenScript). Blots were blocked in 5% BSA made in Tris-buffered saline containing 0.1% Tween 20 (TBST). Blots were incubated overnight at 4 °C with primary antibodies (listed in **Supplementary Table 1**) diluted in TBST (LI-COR Intercept^®^ T20 (TBS) Antibody Diluent (LI-COR, 927–65001)) containing 1% BSA. Following primary incubation, blots were washed and incubated for 2 h at room temperature with near-infrared fluorescent dye-conjugated secondary antibodies (listed in **Supplementary Table 1**) diluted in TBST with 1% BSA. Blots were imaged using LI-COR Odyssey CLx and the densitometric analysis was performed using Image Studio Lite 5.2.5. Image brightness and contrast were uniformly adjusted in Image Studio 4.0.

All original, uncropped immunoblot images used in this study are provided in **Figs. S15-S20**.

### Gel Zymography

The activity of matrix metalloproteinases MMP2 and MMP9 in the ventilation study was assessed by gelatin zymography, and ADAM17 activity by casein zymography, following established protocols^113,114^. Cell culture supernatants from NEG and VENT groups were collected, and protein concentrations were determined by Pierce^™^ BCA^®^ Protein Assay Kit. Equal protein amounts (50 μg) were resolved under non-reducing conditions on 10% polyacrylamide (Sigma-Aldrich, A9099) gels copolymerized with 0.1% (w/v) gelatin (Sigma-Aldrich, G2500) (for MMP 2/9) or 0.1% casein (for ADAM17; Sigma-Aldrich, C8654). After electrophoresis, gels were incubated in renaturation buffer [50 mM Tris-HCl, pH 7.5 (vWR Life Science, 0497 and Fisher Scientific, S25358), 2.5 % (v/v) Triton X-100 (Sigma-Aldrich, T9284), 5 mM CaCl_2_ (Sigma-Aldrich, C4901), 1 mM ZnCl_2_ (Sigma-Aldrich, Z0152)] to restore enzymatic activity, followed by overnight incubation in the same buffer but with 1% (v/v) Triton X-100 at 37 °C. Gels were stained with Coomassie Brilliant Blue R-250 (Sigma-Aldrich, 1125530025), and proteolytic activity was visualized as clear bands against a blue background. Bands corresponding to MMP2, MMP9, and ADAM17 were documented using a Bio-Rad ChemiDoc MP Imaging system and quantified densitometrically with Image Lab software (Bio-Rad, v5.1). Image brightness and contrast were uniformly adjusted in the software. Band intensities were quantified after local background subtraction done through the software. Only linear-range exposures were used for quantification, and adjustments to image brightness/contrast were applied uniformly across the entire stained gels without altering quantitative values. All values represent means from ≥5 independent biological replicates. Normalized intensities were exported from Image Lab and reported as fold-change relative to the indicated control (NEG).

### Immunostaining

AES were retrieved from Mat-loaded devices by enzymatic digestion using 2 mg/mL of Dispase II solution for 1.5 h at 37 °C. Following digestion, AES were gently collected, fixed in 4% PFA for 30 min at room temperature, and washed 3 times with PBS. For cryosectioning, fixed AES were incubated in 30% (w/v) sucrose overnight at 4 °C. Samples were then embedded in optimal cutting temperature cryomatrix and sectioned at 10–15 μm thickness using a Leica CM1950 cryostat at −20 °C. Tissue sections were mounted on positively-charged glass slides for subsequent immunofluorescence staining. Permeabilization was performed with 0.2% Triton X-100 in PBS for 15 min at room temperature. Sections were then blocked with 5% BSA in PBS for 1 h. Primary antibodies, prepared in 1% BSA in PBS at the appropriate dilutions, were added and incubated overnight at 4 °C. The next day, sections were washed 3 times with PBST (PBS containing 0.1% Tween-20) and incubated with fluorophore-conjugated secondary antibodies for 1 h at room temperature. Slides were mounted using VECTASHIELD Antifade Mounting Medium containing DAPI (Vector Laboratories, H-1200–10), and imaging was performed using the Zeiss LSM880 confocal microscope for tissue sections and Leica SP8 DIVE multiphoton microscope for whole mount staining.

For apoptosis detection, Click-iT^™^ Plus TUNEL Assay Kit (Invitrogen, C10618) was used for tissue sections according to the manufacturer’s instructions. TUNEL staining was visualized using the Zeiss Axio Observer fluorescence microscope. Quantification of TUNEL staining was carried out by calculating the percentage of TUNEL-positive fluorescence relative to total DAPI-stained nuclei. The primary and secondary antibodies used, along with their concentrations, are provided in **Supplementary Table 1**.

### 10X Chromium single-cell sequencing

Following dissociation, ATII (P6) single cells of NonBP, 14DPB, and 2 DPI AESs, were flash-frozen at −80 °C until subsequent sequencing. Following thawing, cell number and viability were assessed using a Countess II automated cell counter (Thermo Fisher) and up to 10,000 cells were loaded onto a single lane of a 10X Chromium X. Single cell capture, barcoding, and library preparation were performed using 10X Chromium 3’ RNA v4 according to manufacturer’s protocol. cDNA and library quality were evaluated using 4200 Tapestation (Agilent) and Qubit Fluorometer (Thermo Fisher). Libraries were quantified using KAPA qPCR. The libraries were then sequenced on an Illumina NovaSeq X Plus 200 cycle flow cell lane, targeting 1,500 barcoded cells with an average sequencing depth of 80,000 read pairs per cell.

### Single-cell sequencing analysis

Except where specified, default settings were used for each program. The human ENSEMBL GRCh38.p14 reference assembly and NCBI GenBank Influenza A virus (A/Puerto Rico/8/1934(H1N1)) assembly (GCF_000865725.1) were concatenated and FASTQ files were aligned to the concatenated reference genome using 10X Genomic’s CellRanger (v8.0.1) pipeline.

Except where specified, analysis was performed using Seurat (v5.1.0)^103^. Doublets were identified and removed using scDblFinder (v1.18)^104^. Low-quality cells were removed by filtering for > 100 genes per cell, < 100,000 unique molecular identifiers (UMIs) per cell, and < 20% mitochondrial reads per cell.

Samples were normalized using SCTransform v2 and integrated using HarmonyIntegration. Following integration, the first 35 principal components were used as an input for uniform manifold approximation and projection (UMAP). Differential gene expression was performed using FindMarkers and GO Term enrichment was performed using clusterProfiler^105,106^ and KEGG pathways^107^. Data were analyzed and visualized using the following R packages: Tidyverse^108^, paletteer^109^, ComplexHeatmap^110^, and circlize^111^.

### RNA sequencing

RNA was extracted from the non-infected and infected AES in Trizol using Zymo Direct-zol RNA MiniPrep Kit (Zymo research, R2050) following manufacturer’s protocol. Briefly, frozen cells in Trizol were thawed at room temperature for 5 min, vortexed and equal volume of ethanol was added. After mixing, the solution was transferred to the column for binding, washing, and elution steps. On column DNAase digestion of RNA was carried out using DNAase in the kit. Quantity and RNA integration number (RIN) of the eluted RNA was measured using BioAnalyzer (Agilent Technologies) RNA 6000 Nano Kit. RNA extraction was done in the Penn State College of Medicine Genome Sciences core (RRID:SCR_021123).

RNA-sequencing libraries were prepared in the Penn State College of Medicine Genome Sciences core (RRID:SCR_021123) from 200 ng of total RNA using the KAPA RNA HyperPrep Kits with RiboErase (HMR) (Roche), which targets and depletes both cytoplasmic (5S, 5.8S, 18S, and 28S), and mitochondrial (12S and 16S) ribosomal RNA species from human/mouse/rat with DNA probes and RNase H. The unique dual index sequences (NextFlex Unique Dual Index Barcodes, Perkin Elmer Applied Genomics) were incorporated in the adaptors for multiplexed high-throughput sequencing. Final libraries were assessed for size distribution and concentration using BioAnalyzer High Sensitivity DNA Kit (Agilent Technologies).

The libraries were pooled and sequenced on Illumina NovaSeq 6000 (Illumina), to get on average 25 million, paired end 59 bp reads, according to the manufacturer’s instructions. Samples were demultiplexed using bclconvert software (Illumina). Adaptors were not trimmed during demultiplexing.

FASTQ files were trimmed for adapters and aligned to human genome version hg38 with STAR using default parameters. Raw gene level counts were then obtained by feature Counts using the hg38_ensembl_release108 annotation using hisat2 (v2.1). TheDESeq2 package was utilized to perform normalization of the raw counts. Raw gene counts were then converted to transcripts per million (TPM) and data from 3 independent replicates were analyzed using DESeq2 to obtain differentially expressed genes in infected vs uninfected samples. DESeq2 results were filtered for differentially expressed genes with p-value of less than 0.05 against the MGI database. Ingenuity pathway analysis was used for canonical and upstream pathway analysis of the most highly expressed gene transcripts 1<0.2 as measured by Fisher’s exact test^112^.

### RNA sequencing analysis

Except where specified, analyses were performed in R (v4.3.3). Raw count matrices were log_2_ transformed and descriptive fold-changes were computed between conditions (NonBP vs 1DPB, NonBP vs 10DPB). Gene annotations were mapped from ENSEMBL to HGNC symbols using org.Hs.eg.db (v3.17.0). Gene sets of interest were extracted, z-score normalized, and clustered by Spearman correlations to generate expression heatmaps, with dynamic tree cut clustering applied for unbiased grouping. This was further used in GO enrichment of the obtained cluster-specific signatures using clusterProfiler (v4.8.3) and enrichplot (v1.20.3), with visualization of enriched biological processes exported as dot plots and per-term heatmaps. Selected senescence-associated genes were additionally profiled as robust z-score heatmaps to assess passage-dependent expression dynamics. Differential expression testing between NonBP and 10DPB conditions was performed using limma (v3.56.2), with eBayes moderation of variance. For the GSEA (gene set enrichment analysis), pre-ranked gene lists (by moderated t-statistic) were used for GSEA against GO Biological Process (BP) terms, focusing on processes relevant to epithelial organization, ECM remodeling, and mechanotransduction. For visualization of differentially expressed genes, custom volcano plots were generated (NonBP vs 10DPB) using ggplot2 with significance thresholds set at p < 0.05 and |log_2_FC| > 1. Genes of developmental or mechanotransductive relevance were highlighted by label overlays. Up and downregulated gene lists were exported for further interpretation.

### Statistical analysis

All data were presented as mean ± standard deviation from at least 3 biological replicates or otherwise stated in the manuscript and analyzed by GraphPad Prism 8.4.2 and R 4.4.3. Multiple comparisons were analyzed by one-way analysis of variance (ANOVA) followed by post-hoc Tukey’s multiple-comparison test to determine the individual differences among groups. For comparisons between two different experimental groups, statistical significance was analyzed using two-sided t-tests. Figs. 3e and **3f** present a comparison and analysis of the densitometric quantification of protein expression between NEG and VENT using two-sided paired t-tests. Statistical differences were considered significant at *p < 0.05, **p <0.01, ***p < 0.001. Details of specific statistical methods and *p* value results with the number of independent replicates (*n*) were included within the figures or figure captions.

For bulk-RNA sequencing, raw RNA-seq counts were normalized to reads per kilobase per million mapped reads (RPKM), and log_2_ transformation was applied (log_2_ (RPKM+1) to stabilize variance across samples. Samples were stratified by experimental condition (NonBP, 1 DPB, and 10 DPB), and descriptive log_2_ fold changes were computed between each bioprinted group (1 DPB or 10 DPB) and the non-bioprinted control. For each gene, fold-change was defined as the difference in condition-wise mean log_2_ RPKM. Unsupervised hierarchical clustering of genes of interest (GOI) was performed using both Pearson and Spearman correlation distance metrics and the Ward.D2 linkage method. For functional annotation, the enrichment test used a one-sided hypergeometric test with Benjamini–Hochberg correction for multiple comparisons. A term was considered significantly enriched at false discovery rate (FDR), FDR ≤ 0.05. For visualization, Z-score normalization of expression was performed per gene across all samples, and clustered heatmaps were generated using the pheatmap package. Robust Z-scores were also calculated for all expressed genes to support global variance inspection. Genes associated with senescence and alveolar epithelial differentiation were visualized separately without clustering to preserve biological ordering. All statistical analyses were performed in R (v4.3.3) using open-source Bioconductor packages. Unless otherwise specified, p-values were adjusted for multiple testing using the Benjamini–Hochberg method.

## Supplementary Material

Supplementary Files

This is a list of supplementary files associated with this preprint. Click to download.

- SupplementaryMovie3.mp4
- SupplementaryMovie2.mp4
- SupplementaryMovie1.mp4
- SupplementaryMovie4.mp4
- SupplementaryMovie5.mp4

## References

[1] Fehrenbach H. Alveolar epithelial type II cell: defender of the alveolus revisited. Respiratory research 2, 33 (2001). 11686863 10.1186/rr36 PMC59567

[2] Armstrong L. Expression of functional toll-like receptor-2 and-4 on alveolar epithelial cells. American journal of respiratory cell and molecular biology 31, 241–245 (2004). 15044215 10.1165/rcmb.2004-0078OC

[3] Hogan B. L. Repair and regeneration of the respiratory system: complexity, plasticity, and mechanisms of lung stem cell function. Cell stem cell 15, 123–138 (2014). 25105578 10.1016/j.stem.2014.07.012 PMC4212493

[4] Tata P. R. & Rajagopal J. Plasticity in the lung: making and breaking cell identity. Development 144, 755–766 (2017). 28246210 10.1242/dev.143784 PMC5374348

[5] Nakano S., Hamele C. E., Tata A., Tata P. R. & Heaton N. S. Influenza B virus infection alters the regenerative potential of murine alveolar type 2 pneumocytes. Mbio 16, e02743–02724 (2025). 39745393 10.1128/mbio.02743-24 PMC11796384

[6] Sanders C. J. Compromised respiratory function in lethal influenza infection is characterized by the depletion of type I alveolar epithelial cells beyond threshold levels. American Journal of Physiology-Lung Cellular and Molecular Physiology 304, L481–L488 (2013). 23355384 10.1152/ajplung.00343.2012 PMC3627938

[7] Wernig G. Unifying mechanism for different fibrotic diseases. Proceedings of the National Academy of Sciences 114, 4757–4762 (2017). 10.1073/pnas.1621375114 PMC5422830 28424250

[8] Shiraishi K. Biophysical forces mediated by respiration maintain lung alveolar epithelial cell fate. Cell 186, 1478–1492. e1415 (2023). 36870331 10.1016/j.cell.2023.02.010 PMC10065960

[9] Sucre J. M. Successful establishment of primary type II alveolar epithelium with 3D organotypic coculture. American journal of respiratory cell and molecular biology 59, 158–166 (2018). 29625013 10.1165/rcmb.2017-0442MA PMC6096337

[10] Bluhmki T. Functional human iPSC-derived alveolar-like cells cultured in a miniaturized 96-Transwell air–liquid interface model. Scientific reports 11, 17028 (2021). 34426605 10.1038/s41598-021-96565-4 PMC8382767

[11] Huang J. SARS-CoV-2 infection of pluripotent stem cell-derived human lung alveolar type 2 cells elicits a rapid epithelial-intrinsic inflammatory response. Cell Stem Cell 27, 962–973. e967 (2020). 32979316 10.1016/j.stem.2020.09.013 PMC7500949

[12] Lagowala D. A., Kwon S., Sidhaye V. K. & Kim D.-H. Human microphysiological models of airway and alveolar epithelia. American Journal of Physiology-Lung Cellular and Molecular Physiology 321, L1072–L1088 (2021). 34612064 10.1152/ajplung.00103.2021 PMC8715018

[13] Yadav S. Isogenic induced-pluripotent-stem-cell-derived airway-and alveolus-on-chip models reveal specific innate immune responses. Nature Biomedical Engineering, 1–15 (2025). 10.1038/s41551-025-01444-2 40670718

[14] Si L. A human-airway-on-a-chip for the rapid identification of candidate antiviral therapeutics and prophylactics. Nature biomedical engineering 5, 815–829 (2021). 10.1038/s41551-021-00718-9 PMC8387338 33941899

[15] Jacob A. Derivation of self-renewing lung alveolar epithelial type II cells from human pluripotent stem cells. Nature protocols 14, 3303–3332 (2019). 31732721 10.1038/s41596-019-0220-0 PMC7275645

[16] Al Yazeedi S. The effect of the mechanodynamic lung environment on fibroblast phenotype via the Flexcell. BMC Pulmonary Medicine 24, 362 (2024). 39068387 10.1186/s12890-024-03167-7 PMC11282647

[17] Huang D. Reversed-engineered human alveolar lung-on-a-chip model. Proceedings of the National Academy of Sciences 118, e2016146118 (2021). 10.1073/pnas.2016146118 PMC8126776 33941687

[18] Wei K. Magnetoactive, Kirigami-Inspired Hammocks to Probe Lung Epithelial Cell Function. Cellular and Molecular Bioengineering 17, 317–327 (2024). 39513001 10.1007/s12195-024-00808-z PMC11538102

[19] Ochs M. The number of alveoli in the human lung. American journal of respiratory and critical care medicine 169, 120–124 (2004). 14512270 10.1164/rccm.200308-1107OC

[20] Kawakami M. & Takizawa T. Distribution of pores within alveoli in the human lung. Journal of applied physiology 63, 1866–1870 (1987). 3693221 10.1152/jappl.1987.63.5.1866

[21] Choi Y. m. 3D bioprinted vascularized lung cancer organoid models with underlying disease capable of more precise drug evaluation. Biofabrication 15, 034104 (2023). 10.1088/1758-5090/acd95f 37236168

[22] Sheppard D. Functions of pulmonary epithelial integrins: from development to disease. Physiological reviews 83, 673–686 (2003). 12843406 10.1152/physrev.00033.2002

[23] Nabhan A. N., Brownfield D. G., Harbury P. B., Krasnow M. A. & Desai T. J. Single-cell Wnt signaling niches maintain stemness of alveolar type 2 cells. Science 359, 1118–1123 (2018). 29420258 10.1126/science.aam6603 PMC5997265

[24] Liu S., Sun D., Butler R. & Rawlins E. L. RTK signalling promotes epithelial columnar cell shape and apical junction maintenance in human lung progenitor cells. Development 150 (2023). 10.1242/dev.201284 PMC10281517 37260147

[25] Kaarteenaho R., Merikallio H., Lehtonen S., Harju T. & Soini Y. Divergent expression of claudin-1,−3,−4,−5 and-7 in developing human lung. Respiratory research 11, 59 (2010). 20478039 10.1186/1465-9921-11-59 PMC2886022

[26] Singh J. & Mlodzik M. Planar cell polarity signaling: coordination of cellular orientation across tissues. Wiley Interdisciplinary Reviews: Developmental Biology 1, 479–499 (2012). 23066429 10.1002/wdev.32 PMC3467146

[27] Tilston-Lunel A. M. & Varelas X. in Current Topics in Developmental Biology Vol. 154 285–315 (Elsevier, 2023). 37100521 10.1016/bs.ctdb.2023.02.004

[28] Alysandratos K.-D., Herriges M. J. & Kotton D. N. Epithelial stem and progenitor cells in lung repair and regeneration. Annual review of physiology 83, 529–550 (2021). 10.1146/annurev-physiol-041520-092904 PMC9068227 33074772

[29] Bruewer M., Hopkins A. M., Hobert M. E., Nusrat A. & Madara J. L. RhoA, Rac1, and Cdc42 exert distinct effects on epithelial barrier via selective structural and biochemical modulation of junctional proteins and F-actin. American Journal of Physiology-Cell Physiology 287, C327–C335 (2004). 15044152 10.1152/ajpcell.00087.2004

[30] Lim K. A novel human fetal lung-derived alveolar organoid model reveals mechanisms of surfactant protein C maturation relevant to interstitial lung disease. The EMBO Journal 44, 639–664 (2025). 39815007 10.1038/s44318-024-00328-6 PMC11790967

[31] Hamaratoglu F. The tumour-suppressor genes NF2/Merlin and Expanded act through Hippo signalling to regulate cell proliferation and apoptosis. Nature cell biology 8, 27–36 (2006). 16341207 10.1038/ncb1339

[32] Das T. A molecular mechanotransduction pathway regulates collective migration of epithelial cells. Nature cell biology 17, 276–287 (2015). 25706233 10.1038/ncb3115

[33] Armingol E., Officer A., Harismendy O. & Lewis N. E. Deciphering cell–cell interactions and communication from gene expression. Nature Reviews Genetics 22, 71–88 (2021). 10.1038/s41576-020-00292-x PMC7649713 33168968

[34] Yin F. Spatial organization of Hippo signaling at the plasma membrane mediated by the tumor suppressor Merlin/NF2. Cell 154, 1342–1355 (2013). 24012335 10.1016/j.cell.2013.08.025 PMC3835333

[35] Sabra H. β1 integrin–dependent Rac/group I PAK signaling mediates YAP activation of Yes-associated protein 1 (YAP1) via NF2/merlin. Journal of Biological Chemistry 292, 19179–19197 (2017). 28972170 10.1074/jbc.M117.808063 PMC5702661

[36] Stamenkovic I. & Yu Q. Merlin, a “magic” linker between the extracellular cues and intracellular signaling pathways that regulate cell motility, proliferation, and survival. Current Protein and Peptide Science 11, 471–484 (2010). 20491622 10.2174/138920310791824011 PMC2946555

[37] Mota M. & Shevde L. A. Merlin regulates signaling events at the nexus of development and cancer. Cell Communication and Signaling 18, 1–8 (2020). 32299434 10.1186/s12964-020-00544-7 PMC7164249

[38] Zhang N. The Merlin/NF2 tumor suppressor functions through the YAP oncoprotein to regulate tissue homeostasis in mammals. Developmental cell 19, 27–38 (2010). 20643348 10.1016/j.devcel.2010.06.015 PMC2925178

[39] DiGiovanni G. T. Epithelial Yap/Taz are required for functional alveolar regeneration following acute lung injury. JCI insight 8 (2023). 10.1172/jci.insight.173374 PMC10629815 37676731

[40] Konkimalla A. Multi-apical polarity of alveolar stem cells and their dynamics during lung development and regeneration. Iscience 25 (2022). 10.1016/j.isci.2022.105114 PMC9519774 36185377

[41] Kobayashi Y. Persistence of a regeneration-associated, transitional alveolar epithelial cell state in pulmonary fibrosis. Nature cell biology 22, 934–946 (2020). 32661339 10.1038/s41556-020-0542-8 PMC7461628

[42] Sun Y. L. Heterogeneity in human induced pluripotent stem cell–derived alveolar epithelial type II cells revealed with ABCA3/SFTPC reporters. American Journal of Respiratory Cell and Molecular Biology 65, 442–460 (2021). 34101541 10.1165/rcmb.2020-0259OC PMC8525201

[43] McCauley K. B. Single-cell transcriptomic profiling of pluripotent stem cell-derived SCGB3A2+ airway epithelium. Stem cell reports 10, 1579–1595 (2018). 29657097 10.1016/j.stemcr.2018.03.013 PMC5995784

[44] Zihni C., Mills C., Matter K. & Balda M. S. Tight junctions: from simple barriers to multifunctional molecular gates. Nature reviews Molecular cell biology 17, 564–580 (2016). 27353478 10.1038/nrm.2016.80

[45] Strunz M. Alveolar regeneration through a Krt8+ transitional stem cell state that persists in human lung fibrosis. Nature communications 11, 3559 (2020). 10.1038/s41467-020-17358-3 PMC7366678 32678092

[46] Hansel C., Jendrossek V. & Klein D. Cellular senescence in the lung: the central role of senescent epithelial cells. International journal of molecular sciences 21, 3279 (2020). 32384619 10.3390/ijms21093279 PMC7247355

[47] Werder R. B. CRISPR interference interrogation of COPD GWAS genes reveals the functional significance of desmoplakin in iPSC-derived alveolar epithelial cells. Science Advances 8, eabo6566 (2022). 35857525 10.1126/sciadv.abo6566 PMC9278866

[48] Mathewson A. W., Berman D. G. & Moens C. B. Microtubules are required for the maintenance of planar cell polarity in monociliated floorplate cells. Developmental biology 452, 21–33 (2019). 31029691 10.1016/j.ydbio.2019.04.007 PMC6661169

[49] Ghosh M. C., Makena P. S., Gorantla V., Sinclair S. E. & Waters C. M. CXCR4 regulates migration of lung alveolar epithelial cells through activation of Rac1 and matrix metalloproteinase-2. American Journal of Physiology-Lung Cellular and Molecular Physiology 302, L846–L856 (2012). 22345572 10.1152/ajplung.00321.2011 PMC3362158

[50] Liang C.-L., Li X.-L., Quan X.-J. & Zhang L. DAB2 promotes pulmonary fibrosis and may act as an intermediate between IGF-1R and PI3K/AKT signaling pathways. Experimental and Therapeutic Medicine 25, 183 (2023). 37021069 10.3892/etm.2023.11882 PMC10067542

[51] Ruttkay-Nedecky B. The role of metallothionein in oxidative stress. International journal of molecular sciences 14, 6044–6066 (2013). 23502468 10.3390/ijms14036044 PMC3634463

[52] Sanada F. IGF binding protein-5 induces cell senescence. Frontiers in endocrinology 9, 53 (2018). 29515523 10.3389/fendo.2018.00053 PMC5826077

[53] Kuo M.-L. RRM2B suppresses activation of the oxidative stress pathway and is up-regulated by p53 during senescence. Scientific reports 2, 822 (2012). 23139867 10.1038/srep00822 PMC3492868

[54] Li J. The strength of mechanical forces determines the differentiation of alveolar epithelial cells. Developmental cell 44, 297–312. e295 (2018). 29408236 10.1016/j.devcel.2018.01.008

[55] Gokey J. J. YAP regulates alveolar epithelial cell differentiation and AGER via NFIB/KLF5/NKX2–1. IScience 24 (2021). 10.1016/j.isci.2021.102967 PMC8383002 34466790

[56] Derman I. D. A ventilated perfused lung model platform to dissect the response of the lungs to viral infection. Trends in biotechnology (2025). 10.1016/j.tibtech.2025.03.012 PMC12226239 40280814

[57] Silverthorn U. Human Physiology: An Integrated Approach by Dee. San Fransico: Pearson Benjamin Cummings, 598–601 (2010).

[58] Noto T., Zhou G., Schuele S., Templer J. & Zelano C. Automated analysis of breathing waveforms using BreathMetrics: a respiratory signal processing toolbox. Chemical senses 43, 583–597 (2018). 29985980 10.1093/chemse/bjy045 PMC6150778

[59] van Soldt B. J. & Cardoso W. V. Hippo-Yap/Taz signaling: Complex network interactions and impact in epithelial cell behavior. Wiley Interdisciplinary Reviews: Developmental Biology 9, e371 (2020). 31828974 10.1002/wdev.371 PMC9216164

[60] Ohnishi Y. Screening of factors inducing alveolar type 1 epithelial cells using human pluripotent stem cells. Stem cell reports 19, 529–544 (2024). 38552636 10.1016/j.stemcr.2024.02.009 PMC11096435

[61] Burgess C. L. Generation of human alveolar epithelial type I cells from pluripotent stem cells. Cell Stem Cell 31, 657–675. e658 (2024). 38642558 10.1016/j.stem.2024.03.017 PMC11147407

[62] Sugihara T. YAP tyrosine phosphorylation and nuclear localization in cholangiocarcinoma cells are regulated by LCK and independent of LATS activity. Molecular Cancer Research 16, 1556–1567 (2018). 29903769 10.1158/1541-7786.MCR-18-0158 PMC6170676

[63] Codelia V. A., Sun G. & Irvine K. D. Regulation of YAP by mechanical strain through Jnk and Hippo signaling. Current biology 24, 2012–2017 (2014). 25127217 10.1016/j.cub.2014.07.034 PMC4160395

[64] Ran X. Modulation of the hippo-YAP pathway by cyclic stretch in rat type 2 alveolar epithelial cells—a proof-of-concept study. Frontiers in Physiology 14, 1253810 (2023). 37877098 10.3389/fphys.2023.1253810 PMC10591329

[65] Foda H. D. Ventilator-induced lung injury upregulates and activates gelatinases and EMMPRIN: attenuation by the synthetic matrix metalloproteinase inhibitor, Prinomastat (AG3340). American Journal of Respiratory Cell and Molecular Biology 25, 717–724 (2001). 11726397 10.1165/ajrcmb.25.6.4558f

[66] Dost A. F. A human organoid model of alveolar regeneration reveals distinct epithelial responses to interferon-gamma. bioRxiv, 2025.2001. 2030.635624 (2025).

[67] Toth A. Alveolar epithelial progenitor cells require Nkx2-1 to maintain progenitor-specific epigenomic state during lung homeostasis and regeneration. Nature Communications 14, 8452 (2023). 10.1038/s41467-023-44184-0 PMC10775890 38114516

[68] Zheng M. Mechanosensitive channels in lung disease. Frontiers in Physiology 14, 1302631 (2023). 38033335 10.3389/fphys.2023.1302631 PMC10684786

[69] Zhao B. Inactivation of YAP oncoprotein by the Hippo pathway is involved in cell contact inhibition and tissue growth control. Genes & development 21, 2747–2761 (2007). 17974916 10.1101/gad.1602907 PMC2045129

[70] Konishi S., Tata A. & Tata P. R. Defined conditions for long-term expansion of murine and human alveolar epithelial stem cells in three-dimensional cultures. STAR protocols 3, 101447 (2022). 35712012 10.1016/j.xpro.2022.101447 PMC9192963

[71] Palomino-Segura M. Protection against influenza infection requires early recognition by inflammatory dendritic cells through C-type lectin receptor SIGN-R1. Nature microbiology 4, 1930–1940 (2019). 10.1038/s41564-019-0506-6 PMC6817362 31358982

[72] Herold S. Alveolar epithelial cells direct monocyte transepithelial migration upon influenza virus infection: impact of chemokines and adhesion molecules. The Journal of Immunology 177, 1817–1824 (2006). 16849492 10.4049/jimmunol.177.3.1817

[73] Stegemann-Koniszewski S. Alveolar type II epithelial cells contribute to the anti-influenza A virus response in the lung by integrating pathogen-and microenvironment-derived signals. MBio 7, 10.1128/mbio.00276-00216 (2016). PMC4959657 27143386

[74] Lindell D. M., Standiford T. J., Mancuso P., Leshen Z. J. & Huffnagle G. B. Macrophage inflammatory protein 1α/CCL3 is required for clearance of an acute Klebsiella pneumoniae pulmonary infection. Infection and immunity 69, 6364–6369 (2001). 11553580 10.1128/IAI.69.10.6364-6369.2001 PMC98771

[75] Wang J. Differentiated human alveolar type II cells secrete antiviral IL-29 (IFN-λ1) in response to influenza A infection. The Journal of Immunology 182, 1296–1304 (2009). 19155475 10.4049/jimmunol.182.3.1296 PMC4041086

[76] Coates B. M. Inflammatory monocytes drive influenza A virus–mediated lung injury in juvenile mice. The Journal of Immunology 200, 2391–2404 (2018). 29445006 10.4049/jimmunol.1701543 PMC5860989

[77] Killip M. J., Fodor E. & Randall R. E. Influenza virus activation of the interferon system. Virus research 209, 11–22 (2015). 25678267 10.1016/j.virusres.2015.02.003 PMC4638190

[78] Zhu Y., Song D., Song Y. & Wang X. Interferon gamma induces inflammatory responses through the interaction of CEACAM1 and PI3K in airway epithelial cells. Journal of translational medicine 17, 147 (2019). 31072323 10.1186/s12967-019-1894-3 PMC6507156

[79] Schneider C. Alveolar macrophages are essential for protection from respiratory failure and associated morbidity following influenza virus infection. PLoS pathogens 10, e1004053 (2014). 24699679 10.1371/journal.ppat.1004053 PMC3974877

[80] Yoshida M., Ikegami M., Reed J. A., Chroneos Z. C. & Whitsett J. A. GM-CSF regulates protein and lipid catabolism by alveolar macrophages. American Journal of Physiology-Lung Cellular and Molecular Physiology 280, L379–L386 (2001). 11159019 10.1152/ajplung.2001.280.3.L379

[81] Sever-Chroneos Z. GM-CSF modulates pulmonary resistance to influenza A infection. Antiviral research 92, 319–328 (2011). 21925209 10.1016/j.antiviral.2011.08.022 PMC4894852

[82] Halstead E. S. GM-CSF overexpression after influenza a virus infection prevents mortality and moderates M1-like airway monocyte/macrophage polarization. Respiratory Research 19, 3 (2018). 29304863 10.1186/s12931-017-0708-5 PMC5756339

[83] Ito Y. Influenza induces IL-8 and GM-CSF secretion by human alveolar epithelial cells through HGF/c-Met and TGF-α/EGFR signaling. American Journal of Physiology-Lung Cellular and Molecular Physiology 308, L1178–L1188 (2015). 26033355 10.1152/ajplung.00290.2014 PMC4451400

[84] Huang F.-F. GM-CSF in the lung protects against lethal influenza infection. American journal of respiratory and critical care medicine 184, 259–268 (2011). 21474645 10.1164/rccm.201012-2036OC PMC6938174

[85] Gu Y. The mechanism behind influenza virus cytokine storm. Viruses 13, 1362 (2021). 34372568 10.3390/v13071362 PMC8310017

[86] Williams T. C. IL-25 blockade augments antiviral immunity during respiratory virus infection. Communications Biology 5, 415 (2022). 35508632 10.1038/s42003-022-03367-z PMC9068710

[87] Hong H. Induction of IL-25 expression in human nasal polyp epithelium by influenza virus infection is abated by interferon-alpha pretreatment. Journal of Inflammation Research, 2769–2780 (2021). 34234504 10.2147/JIR.S304320 PMC8254189

[88] Sun K., Torres L. & Metzger D. W. A detrimental effect of interleukin-10 on protective pulmonary humoral immunity during primary influenza A virus infection. Journal of virology 84, 5007–5014 (2010). 20200252 10.1128/JVI.02408-09 PMC2863832

[89] Bridges J. P. LPCAT1 regulates surfactant phospholipid synthesis and is required for transitioning to air breathing in mice. The Journal of clinical investigation 120, 1736–1748 (2010). 20407208 10.1172/JCI38061 PMC2860922

[90] Barkauskas C. E. Type 2 alveolar cells are stem cells in adult lung. The Journal of clinical investigation 123, 3025–3036 (2013). 23921127 10.1172/JCI68782 PMC3696553

[91] Jacob A. Differentiation of human pluripotent stem cells into functional lung alveolar epithelial cells. Cell stem cell 21, 472–488. e410 (2017). 28965766 10.1016/j.stem.2017.08.014 PMC5755620

[92] Weiner A. I. Mesenchyme-free expansion and transplantation of adult alveolar progenitor cells: steps toward cell-based regenerative therapies. NPJ Regenerative medicine 4, 17 (2019). 31452939 10.1038/s41536-019-0080-9 PMC6702233

[93] Brassard J. A., Nikolaev M., Hübscher T., Hofer M. & Lutolf M. P. Recapitulating macro-scale tissue self-organization through organoid bioprinting. Nature Materials 20, 22–29 (2021). 32958879 10.1038/s41563-020-00803-5

[94] Wheaton A. K., Agarwal M., Jia S. & Kim K. K. Lung epithelial cell focal adhesion kinase signaling inhibits lung injury and fibrosis. American Journal of Physiology-Lung Cellular and Molecular Physiology 312, L722–L730 (2017). 28283477 10.1152/ajplung.00478.2016 PMC5451599

[95] Hicks-Berthet J. & Varelas X. Integrin-FAK-CDC42-PP1A signaling gnaws at YAP/TAZ activity to control incisor stem cells. Bioessays 39, 1700116 (2017). 10.1002/bies.201700116 PMC5659350 28891248

[96] Slepushkin V. A., Staber P. D., Wang G., McCray P. B. & Davidson B. L. Infection of human airway epithelia with H1N1, H2N2, and H3N2 influenza A virus strains. Molecular Therapy 3, 395–402 (2001). 11273782 10.1006/mthe.2001.0277 PMC7106098

[97] Maginnis M. S. Virus–receptor interactions: the key to cellular invasion. Journal of molecular biology 430, 2590–2611 (2018). 29924965 10.1016/j.jmb.2018.06.024 PMC6083867

[98] Ivanova P. T. Lipid Composition of the Viral Envelope of Three Strains of Influenza Virus Not All Viruses Are Created Equal. ACS infectious diseases 1, 435–442 (2015). 10.1021/acsinfecdis.5b00040 PMC4593503 26448476

[99] Miao Y. Co-development of mesoderm and endoderm enables organotypic vascularization in lung and gut organoids. Cell (2025). 10.1016/j.cell.2025.05.041 PMC13056595 40592324

[100] Kim M. H. & Ozbolat I. T. Aspiration-assisted bioprinting of spheroids. Nature Protocols, 1–49 (2025). 40973814 10.1038/s41596-025-01240-x PMC13005709

[101] Co J. Y., Margalef-Català M., Monack D. M. & Amieva M. R. Controlling the polarity of human gastrointestinal organoids to investigate epithelial biology and infectious diseases. Nature protocols 16, 5171–5192 (2021). 34663962 10.1038/s41596-021-00607-0 PMC8841224

[102] Hao Y. Dictionary learning for integrative, multimodal and scalable single-cell analysis. Nature biotechnology 42, 293–304 (2024). 10.1038/s41587-023-01767-y PMC10928517 37231261

[103] Germain P.-L., Lun A., Meixide C. G., Macnair W. & Robinson M. D. Doublet identification in single-cell sequencing data using scDblFinder. f1000research 10, 979 (2022). 10.12688/f1000research.73600.1 PMC9204188 35814628

[104] Yu G., Wang L.-G., Han Y. & He Q.-Y. clusterProfiler: an R package for comparing biological themes among gene clusters. Omics: a journal of integrative biology 16, 284–287 (2012). 22455463 10.1089/omi.2011.0118 PMC3339379

[105] Wu T. clusterProfiler 4.0: A universal enrichment tool for interpreting omics data. The innovation 2 (2021). 10.1016/j.xinn.2021.100141 PMC8454663 34557778

[106] Kanehisa M., Furumichi M., Sato Y., Matsuura Y. & Ishiguro-Watanabe M. KEGG: biological systems database as a model of the real world. Nucleic acids research 53, D672–D677 (2025). 39417505 10.1093/nar/gkae909 PMC11701520

[107] Wickham H. Welcome to the Tidyverse. Journal of open source software 4, 1686 (2019).

[108] Hvitfeldt E. paletteer: Comprehensive Collection of Color Palettes. (2021).

[109] Gu Z. Complex heatmap visualization. Imeta 1, e43 (2022). 38868715 10.1002/imt2.43 PMC10989952

[110] Gu Z., Gu L., Eils R., Schlesner M. & Brors B. “ Circlize” implements and enhances circular visualization in R. (2014). 10.1093/bioinformatics/btu393 24930139

[111] Yau E. Genomic and epigenomic adaptation in SP-R210 (Myo18A) isoform-deficient macrophages. Immunobiology 226, 152150, doi:10.1016/j.imbio.2021.152150 (2021). 34735924 PMC8863115

[112] Tajhya R. B., Patel R. S. & Beeton C. in Matrix Metalloproteases: Methods and Protocols 231–244 (Springer, 2017).

[113] Kennedy C. C., Kottom T. J. & Limper A. H. Characterization of a novel ADAM protease expressed by Pneumocystis carinii. Infection and immunity 77, 3328–3336 (2009). 19451239 10.1128/IAI.01383-08 PMC2715687

